# Pax6 Exerts Regional Control of Cortical Progenitor Proliferation via Direct Repression of *Cdk6* and Hypophosphorylation of pRb

**DOI:** 10.1016/j.neuron.2013.02.012

**Published:** 2013-04-24

**Authors:** Da Mi, Catherine B. Carr, Petrina A. Georgala, Yu-Ting Huang, Martine N. Manuel, Emily Jeanes, Emi Niisato, Stephen N. Sansom, Frederick J. Livesey, Thomas Theil, Kerstin Hasenpusch-Theil, T. Ian Simpson, John O. Mason, David J. Price

**Affiliations:** 1 Centre for Integrative Physiology, University of Edinburgh, Edinburgh EH8 9XD, UK; 2 The Gurdon Institute, University of Cambridge, Cambridge CB2 1QN, UK

## Abstract

The mechanisms by which early spatiotemporal expression patterns of transcription factors such as Pax6 regulate cortical progenitors in a region-specific manner are poorly understood. Pax6 is expressed in a gradient across the developing cortex and is essential for normal corticogenesis. We found that constitutive or conditional loss of Pax6 increases cortical progenitor proliferation by amounts that vary regionally with normal Pax6 levels. We compared the gene expression profiles of equivalent *Pax6*-expressing progenitors isolated from *Pax6*^*+/+*^ and *Pax6*^*−/−*^ cortices and identified many negatively regulated cell-cycle genes, including *Cyclins* and *Cdks*. Biochemical assays indicated that Pax6 directly represses *Cdk6* expression. Cyclin/Cdk repression inhibits retinoblastoma protein (pRb) phosphorylation, thereby limiting the transcription of genes that directly promote the mechanics of the cell cycle, and we found that Pax6 inhibits pRb phosphorylation and represses genes involved in DNA replication. Our results indicate that Pax6’s modulation of cortical progenitor cell cycles is regional and direct.

## Highlights

• Pax6 loss increases cortical progenitor proliferation by region-specific amounts
• The size of this effect correlates directly with normal Pax6 expression levels
• Expression of many key cell-cycle regulators is increased in the absence of Pax6
• Pax6 directly represses Cdk6 expression and controls pRb phosphorylation

## Teaser

The transcription factor Pax6 regulates multiple aspects of forebrain development. Mi et al. show that it exerts regional control of cortical cell proliferation. It inhibits cell-cycle gene expression, directly repressing Cdk6, and limits the activity of the pRb-E2F pathway.

## Introduction

Understanding how transcription factors that regulate specific spatiotemporal patterns of gene expression control cellular development remains a major challenge. Although many transcription factors essential for tissue specification have been discovered and cellular processes that they influence have been identified, how they operate is largely unknown. Current evidence indicates that these transcription factors control large gene regulatory networks comprising many, possibly thousands, of target genes, some of which encode other transcription factors (Biggin, 2011). The number of interactions separating a transcription factor from its cellular effects is, therefore, potentially extremely large, and the cellular processes that it regulates may be a long way downstream of its primary molecular actions. It is important to establish how directly any given transcription factor might influence the cellular processes that it regulates, to provide a framework for building a more detailed understanding of its mechanisms of action.

Pax6 is one of the best-studied high-level transcription factors regulating development and implicated in disease. In vertebrates, most Pax6 expression is restricted to specific populations of central nervous system (CNS) progenitor cells, including those of the mammalian cerebral cortex (Manuel and Price, 2005; Georgala et al., 2011b). In humans, mutations of *PAX6* result in eye defects and neurological abnormalities linked to structural alterations of the brain (Sisodiya et al., 2001). PAX6 has also been implicated as a tumor suppressor in human gliomas (Appolloni et al., 2012). Mice with *Pax6* loss-of-function mutations show eye and CNS defects (Hill et al., 1991; Stoykova et al., 1996; Manuel and Price, 2005; Georgala et al., 2011b). Several studies have implicated Pax6 in the regulation of neural progenitor proliferation, but the nature and significance of this regulation are poorly understood and its mechanism is unknown (Estivill-Torrus et al., 2002, Manuel et al., 2007, Georgala et al., 2011a; Asami et al., 2011). Through a region-specific action on cell proliferation, Pax6 may influence many aspects of brain development, including major features such as regional differences in the size and shape of brain structures.

Our first aim was to examine the nature and significance of Pax6’s regulation of cortical progenitor proliferation by examining mouse models with either complete or conditional loss of Pax6 function. Previous studies have suggested that gradients of Pax6 expression present across the cortex at early stages of corticogenesis are important for its regionalization in terms of later differentiation (Bishop et al., 2000), but whether these gradients cause regional effects on early proliferation is unclear. Moreover, the effects on proliferation of changes in these Pax6-expression gradients with age have not been explored.

Our second aim was to explore the mechanisms by which Pax6 might regulate cortical progenitor proliferation. To do this, we used a more focused approach than that employed in previous screens aimed at identifying Pax6-regulated genes (Holm et al., 2007; Sansom et al., 2009). To screen for genes whose expression levels in *Pax6*-expressing cortical progenitors depend on whether these cells express Pax6 protein or not, we isolated *Pax6*-expressing progenitors using a line of reporter mice carrying a YAC transgene (*DTy54*) that expresses GFP under the control of *PAX6*’s regulatory elements (Tyas et al., 2006) irrespective of the status of the endogenous *Pax6* locus. In these mice, GFP is expressed by *Pax6*-expressing cortical progenitors but not by the postmitotic neurons they give rise to (Tyas et al., 2006), allowing us to compare profiles of gene expression in equivalent populations.

The discoveries we made led us to examine Pax6’s regulation of the expression of the cyclin-dependent kinase Cdk6. In mammals, the Cdks and their partners the cyclins are the primary regulators of transition through the cell cycle (Malumbres and Barbacid, 2005). D-type cyclins facilitate the progression of progenitors, including cortical progenitors, through G1, a critical stage that allows responses to signals inducing either commitment to further stages of the cell cycle or withdrawal from the cell cycle (Zetterberg et al., 1995; Glickstein et al., 2009; Dehay and Kennedy, 2007; Lange et al., 2009; Pilaz et al., 2009). Once they are associated with cyclins, activated Cdks phosphorylate downstream targets needed for progression through the cell cycle, including retinoblastoma protein (pRb), resulting in the disassociation of pRb/E2F complexes and promotion of the G1 to S phase transition (Ferguson and Slack, 2001; Harbour et al., 1999; Di Stefano et al., 2003; Lee et al., 2000; Polager and Ginsberg, 2008; Goto et al., 2006; Malumbres and Barbacid, 2005). We found evidence that this pathway is directly regulated by Pax6.

## Results

### Pax6 Slows Progenitor Cell-Cycle Times in Regions of Embryonic Day 12.5 Cortex where its Expression Is Highest

**Figure 1:**
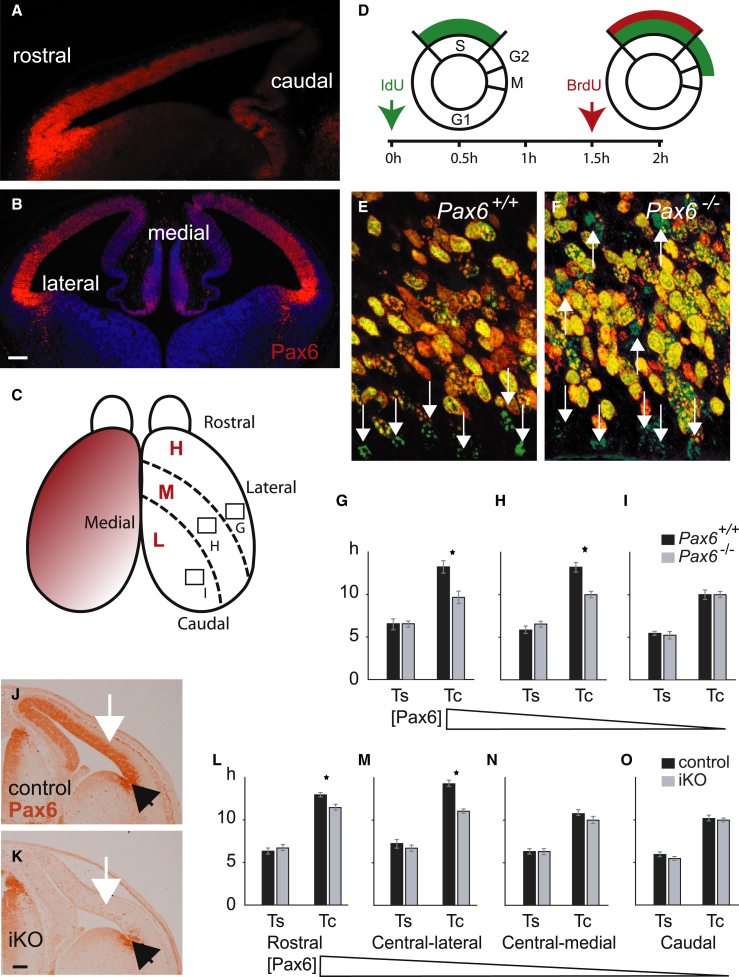
Absence of Pax6 Causes Cell-Cycle Acceleration in Cortical Areas that Normally Express Most Pax6 at E12.5 (A–C) Pax6 protein (red) is normally expressed in a ^high^rostrolateral to ^low^caudomedial gradient at E12.5. For analysis of cell-cycle times in *Pax6*^*−/−*^ mutants, measurements were made in three zones (G, central-lateral; H, central-medial; I, caudal), expressing high (H), medium (M), or low (L) levels of Pax6, respectively. Scale bars represent 100 μm (A and B). (D) Cell-cycle times at E12.5 were calculated as in Martynoga et al. (2005) from counts of labeled cells obtained by injecting pregnant dams with IdU followed 1.5 hr later by BrdU. (E and F) Examples of cells from *Pax6*^*+/+*^ and *Pax6*^*−/−*^ embryos labeled with IdU alone (green; white arrows) or with both BrdU (red) and IdU. In area G in (C) there are more labeled cells in total and more single-IdU-labeled cells in mutants. (G–I) Means (±SEM) for lengths of S phase (Ts) and the overall cell cycle (Tc) in each of the three areas marked in (C) in *Pax6*^*+/+*^ and *Pax6*^*−/−*^ embryos. (G) n = 4 embryos per genotype, ^∗^p < 0.03, Student’s t test. n = 3 (H and I), ^∗^p < 0.05. (J and K) Loss of Pax6 protein from E12.5 cortex (white arrows) induced by tamoxifen given at E9.5 to *Pax6*^*loxP*/*loxP*^; *Emx1-CreER*^T2^ (iKO) embryos. Note the retention of Pax6 in the ventral pallium (black arrows), where *Emx1* is not expressed (scale bar, 100 μm). (L–O) Mean Ts and Tc (±SEM; calculated as in D–F) in iKOs (as in J and K) in four regions of the cortex with progressively lower Pax6 levels (n = 3 embryos per genotype, ^∗^p < 0.0001, Sidak’s multiple-comparisons test). See also Figure S1.

At the onset of corticogenesis, around embryonic day 12.5 (E12.5), Pax6 is expressed in a gradient across the embryonic cortex with high levels rostrolaterally and low levels caudomedially (Figures 1A–1C; Bishop et al., 2000; Manuel et al., 2007). We used iododeoxyuridine (IdU) and bromodeoxyuridine (BrdU) double labeling as summarized in Figure 1D to calculate the cell-cycle and S phase times (Tc and Ts, respectively; Martynoga et al., 2005; Figures 1E and 1F) in regions of E12.5 *Pax6*^*+/+*^ and *Pax6*^*−/−*^ cortex expressing high, medium, or low levels of Pax6 (Figures 1C and 1G–1I). In *Pax6*^*+/+*^ embryos, the mean Tc was longest in areas expressing high or medium levels of Pax6 (Figures 1G–1I). In *Pax6*^*−/−*^ embryos, the mean Tc was significantly shorter by ∼25% in these two areas. Neither the mean Tc in the area of lowest Pax6 expression nor the mean Ts in any area was affected in *Pax6*^*−/−*^ embryos.

We also tested the effect of an acute (conditional) loss of Pax6, since the rapid onset of a defect would strengthen the possibility that Pax6 influences the cell cycle directly. We analyzed mice carrying a tamoxifen-induced, cortex-specific deletion of *Pax6*. Their genotypes were as follows: (1) *Pax6*^*loxP*/*loxP*^; *Emx1-CreER*^T2^; *R26R-YFP* (inducible knockout [iKO] embryos); and (2) *Pax6*^*loxP*/*+*^; *Emx1-CreER*^T2^; *R26R-YFP* embryos (controls; deletion of only one copy of Pax6 has no detectable effect on cortical progenitor proliferation; Figure S1 available online). Tamoxifen administered at E9.5 resulted in loss of Pax6 from the cortex (sparing the ventral pallium, which does not express *Emx1*) by E12.5 (Figures 1J and 1K). The results obtained from iKOs were similar to those from *Pax6*^*−/−*^ embryos, with significant effects of both genotype and cortical region on mean Tc (two-way ANOVA). In rostral and central-lateral areas (i.e., [Pax6]^high^ in controls), the mean Tc was longer than in central-medial and caudal areas in controls (p < 0.0001, Sidak’s multiple-comparisons test) and was significantly reduced in iKOs (Figures 1L–1O). These results show an association between the spatial distribution of Pax6 across the cortex and both progenitor Tc in wild-types (WTs) and the effect of Pax6 absence on Tc in mutants at E12.5.

### Deletion of Pax6 Has More Widespread Effects on Proliferation at Later Embryonic Ages, when Its Expression Is More Uniform

**Figure 2:**
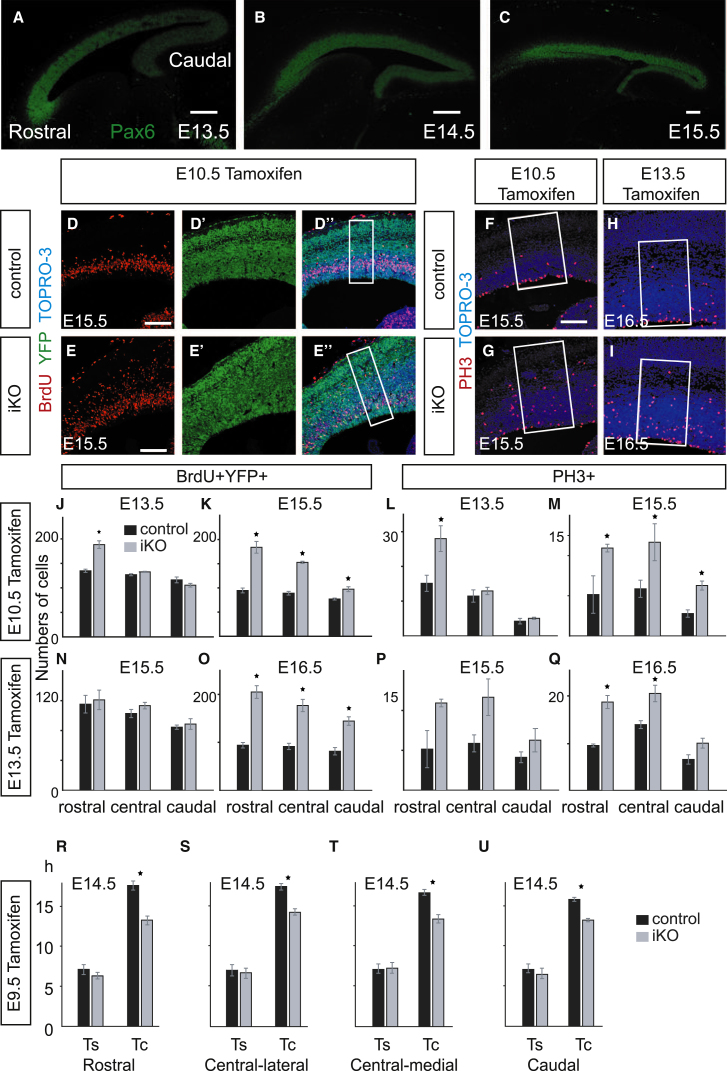
Loss of Pax6 Causes Increasingly Widespread Rapid-Onset Proliferative Defects after E13.5 (A–C) Expression of Pax6 protein in parasagittal sections from E13.5–E15.5 WT embryos (all oriented the same way; scale bars, 100 μm). (D–E″) BrdU and YFP immunohistochemistry on brain sections from E15.5 iKO and control embryos after tamoxifen administration on E10.5. (D and E) More BrdU-labeled cells were present in iKO cortex. (D′ and E′) Almost all cortical cells were YFP positive. (D″ and E″) Merged images: BrdU-positive cells were counted in radially arranged 100-μm-wide boxes, as illustrated. (F–I) PH3 immunohistochemistry on sections from E15.5 and E16.5 iKO and control embryos after tamoxifen administration on E10.5 or E13.5. Increased numbers of PH3-positive cells were observed in iKOs. PH3-positive cells were counted in 200-μm-wide boxes, as illustrated. Scale bars = 100 μm. (J–Q) Average numbers (±SEM) of BrdU-YFP double-labeled cells or nonapical PH3-labeled cells in counting boxes (see D”, E”, and F–I) from rostral, central, and caudal cortical areas in E13.5, E15.5, or E16.5 iKO or control embryos injected with tamoxifen on E10.5 or E13.5. (J) ^∗^p < 0.003, Student’s t test. (K) ^∗^p < 0.002 rostrally, ^∗^p < 0.001 centrally, ^∗^p < 0.02 caudally. (L) ^∗^p < 0.04. (M) ^∗^p < 0.001 rostrally, ^∗^p < 0.002 centrally, ^∗^p < 0.001 caudally. (O) ^∗^p < 0.002 rostrally, ^∗^p < 0.004 centrally, ^∗^p < 0.006 caudally. (Q) ^∗^p < 0.003 rostrally, ^∗^p < 0.03 centrally. In all cases, n = 3 embryos per genotype. (R–U) Measurements of mean Ts and Tc (±SEM) in four cortical areas in control and iKO E14.5 embryos after tamoxifen administration at E9.5 (n = 3 embryos per genotype; ^∗^p < 0.0001, Sidak’s multiple-comparisons test). See also Figures S2 and S3.

The expression of Pax6 across the cortex becomes increasingly uniform with embryonic age, with similar levels being attained in all areas by E15.5 (Figures 2A–2C), suggesting that the regional effects of Pax6 loss on progenitor proliferation might be different at these ages. In a first set of experiments, iKOs were generated by tamoxifen administration at either E10.5 or E13.5 (Figures 2D–2Q). Activation of YFP from the *R26R-YFP* allele occurred within 48 hr (Figure S2A). Quantitative RT-PCR (qRT-PCR) on cortical messenger RNA (mRNA) showed that *Pax6* levels in iKOs had fallen to 36.5% (±7.9 SEM; n = 3 independent experiments) of control values within 48 hr of E10.5 tamoxifen administration, and to 9.8% (±7.0; n = 3) and 1.6% (±0.6; n = 3) by 72 hr and 96 hr, respectively. Immunohistochemistry showed no obvious loss of Pax6 protein from the cortex 48 hr after tamoxifen administration (Figures S2B and S2F), presumably due to residual protein perdurance. Within 72 hr of tamoxifen administration, however, Pax6 protein was removed from most cells in *Emx1*’s cortical expression domain (Figures S2C, S2G, S2E, and S2I).

We compared the numbers of YFP-positive cells in S phase in rostral, central, and caudal areas of the cortex (high, medium, and low Pax6-expressing, respectively; Figures 2D–2E″, 2J, 2K, 2N, and 2O) in iKO and control embryos. Most cortical cells were YFP labeled in these embryos (Figures 2D’ and 2E’), and the proportions that were not ranged from 5% to 15% in both iKO and control cortices. Cells in S phase were identified by a 1 hr pulse of BrdU. The average numbers of YFP-positive cells that were in S phase at different times after tamoxifen administration at E10.5 (iKO^E10.5tamox^) or E13.5 (iKO^E13.5tamox^) are shown in Figures 2J, 2K, 2N, and 2O. In E13.5 iKO^E10.5tamox^ embryos, i.e., shortly after loss of almost all Pax6 protein, increases in the numbers of cells in S phase occurred specifically in the rostral cortex (Figure 2J), indicating rapid onset of overproliferation confined to this region. Two days later, however, in E15.5 iKO^E10.5tamox^ embryos, significant increases in the number of cells in S phase were found in all parts of the cortex (Figure 2K). Similarly, between E15.5 and E16.5 in iKO^E13.5tamox^ embryos, significant increases in the number of cells in S phase occurred in all cortical areas (Figures 2N and 2O).

In a second set of experiments, we estimated (as in Figures 1D–1F) values for mean Tc and Ts in iKO^E9.5tamox^ embryos at E14.5 (Figures 2R–2U; Figures S2D and S2H). The mean Tc varied significantly with genotype and cortical area (two-way ANOVA). It was reduced significantly in all cortical areas in iKOs (Figures 2R–2U). In controls, the mean Tc was slightly lower in the caudal cortex than in the rostral and central-lateral cortex (p < 0.0003 and < 0.015, respectively; Sidak’s multiple-comparisons test). The mean Ts did not show differences with genotype or cortical area. These results indicate that loss of Pax6 causes shortening of the cell cycle across all cortical areas by E14.5.

Given that Tcs are shortened after loss of Pax6, either in specific cortical regions or across the entire cortex, depending on age, we predicted that these changes should correlate with an increased incidence of cells in M phase (identified by their expression of phosphorylated histone 3 [PH3]). This proved to be true (Figures 2F–2I, 2L, 2M, 2P, and 2Q; Figure S3). Interestingly, the positions of these additional M phase cells were abnormal. The increases were invariably nonapical, i.e., they occurred through the depth of the ventricular zone rather than at the ventricular surface, where most mitoses normally occur (Figures 2F–2I; Figure S3). This phenomenon was previously described in *Pax6*^*−/−*^ embryos (Estivill-Torrus et al., 2002; Quinn et al., 2007; Asami et al., 2011). It may be due to a breakdown in coordination between interkinetic migration and mitosis as a result of cell-cycle shortening rather than representing an expanded mutant equivalent of the intermediate progenitor population, since the numbers of cells expressing the classical marker of intermediate progenitors, Tbr2, are greatly reduced in *Pax6*^*−/−*^ cortices (Quinn et al., 2007). We found that in E13.5 iKO^E10.5tamox^ embryos, the numbers of M phase cells were significantly increased only in the rostral cortex (Figure 2L), but 2 days later, in E15.5 iKO^E10.5tamox^ embryos, they were significantly increased in all parts of the cortex (Figure 2M). In iKO^E13.5tamox^ embryos, significant increases in the numbers of M phase cells occurred between E15.5 and E16.5 in both the central and rostral cortex, but not in the caudal cortex (Figures 2P and 2Q).

The results of these experiments indicate that during corticogenesis, Pax6 exerts a repressive action on the proliferation of progenitors, and the dynamics of the Pax6 expression gradient and rates of progenitor proliferation are correlated. At E12.5, when the Pax6 gradient is steepest, areas of highest expression correlate with regions where Tc is longest. Loss of Pax6 causes shortening of Tc only in these areas. In normal embryos at older ages, the Pax6 gradient becomes progressively more uniform across the cortex, as does Tc, and loss of Pax6 causes shortening of Tc in all areas.

### Pax6 Regulates the Expression of Cell-Cycle Genes in Cortical Progenitors

To investigate the molecular mechanisms by which Pax6 regulates cortical progenitor cell proliferation, we first identified cell-cycle genes with altered expression levels in progenitor cells in *Pax6*^*−/−*^ mutants. To do this, we generated litters of mice containing *Pax6*^*−/−*^ and *Pax6*^*+/+*^ E12.5 embryos that also carried the *DTy54* transgene. This GFP reporter can be used to distinguish cells in which the endogenous *Pax6* locus is transcriptionally active irrespective of whether it contains a WT or mutant allele (Tyas et al., 2006), allowing comparison of gene expression in equivalent *Pax6*-expressing progenitors from *Pax6*^*+/+*^ versus *Pax6*^*−/−*^ cortices. The expression of GFP (Figures S4A–S4D) provided a guide for the dissection of regions of cortex with the highest *Pax6* gene expression.

Cells from these regions were dissociated and *Pax6*-expressing cells were obtained by fluorescence-activated cell sorting (FACS; Figures S4E–S4H). The gate was set to include only those cells with GFP fluorescence greater than that of all cells in samples from non-*DTy54*-carrying controls, so as to enrich for *Pax6*-expressing progenitors by including only those cells with the highest GFP levels. We obtained similar proportions of *Pax6*^*+/+*^;*DTy54*^*+*^ and *Pax6*^*−/−*^;*DTy54*^*+*^ cells with fluorescence levels within the gate.

We carried out three independent experiments, each of which used samples of mRNA from a different pool of *Pax6*^*+/+*^ and *Pax6*^*−/−*^ littermates obtained on different occasions and with RNA integrity (RIN) scores ≥ 9.3/10 for Agilent microarray comparisons. In a preliminary statistical analysis of the normalized data, we assigned a p value to the change in expression of each gene using an empirical Bayes moderated t test (Efron and Tibshirani, 2002). These p values were adjusted to correct for multiple t tests (Benjamini and Hochberg, 1995). We identified 411 genes whose expression was significantly (i.e., adjusted p value < 0.05) upregulated and 532 genes whose expression was significantly downregulated. Full data sets are provided on the Gene Expression Omnibus website (http://www.ncbi.nlm.nih.gov/geo/query/acc.cgi?acc=GSE38703). Further quality control assessments are included in Figures S5A–S5D.

**Figure 3:**
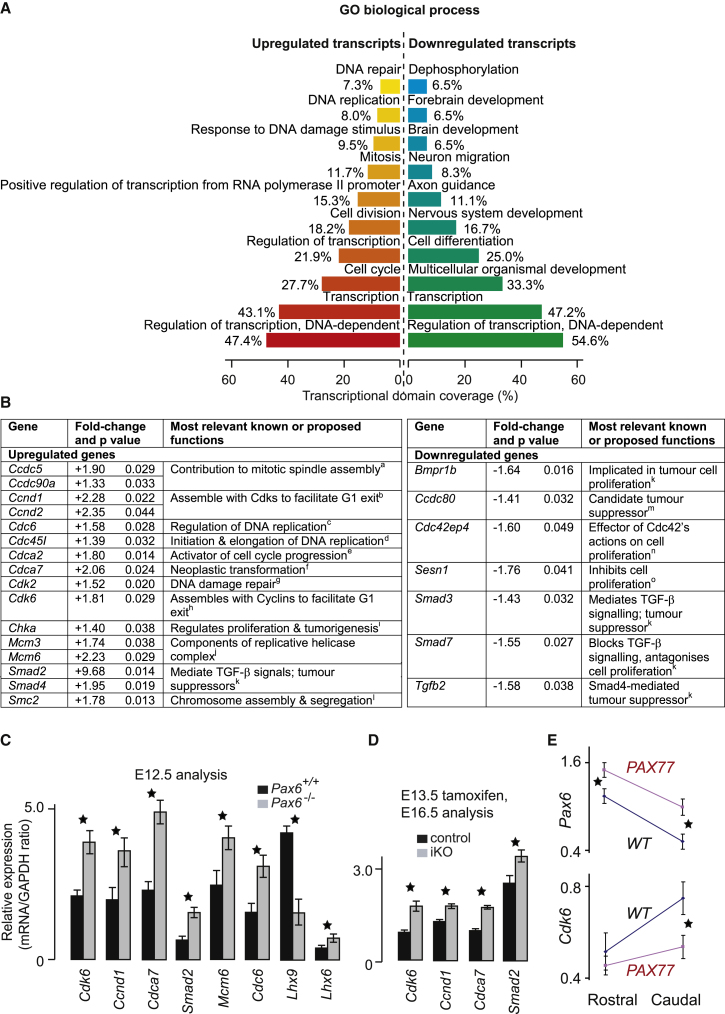
Loss of Pax6 Results in Numerous Changes in Expression of Cell-Cycle Genes (A) The top ten overrepresented biological processes identified by GO analysis of all genes with a significant (p < 0.05) increase or decrease in expression. Transcriptional domain coverage is the number of transcripts in each significantly overrepresented theme expressed as a percentage of all transcripts annotated by at least one significantly overrepresented theme. (B) Cell-cycle genes whose expression levels were significantly changed in the absence of Pax6 (^a^Einarson et al., 2004; ^b^Sherr, 1995; ^c^Knockleby and Lee, 2010; ^d^Tomita et al., 2011; ^e^Ryu et al., 2007; ^f^Goto et al., 2006; ^g^Satyanarayana and Kaldis, 2009; ^h^Tashiro et al., 2007; ^i^Miyake and Parsons, 2012; ^j^Bochman and Schwacha, 2009; ^k^Meulmeester and Ten Dijke, 2011; ^l^Losada and Hirano, 2005; ^m^Bommer et al., 2005; ^n^Stengel and Zheng, 2011; ^o^Budanov and Karin, 2008). The p values were obtained after adjustment (Benjamini and Hochberg, 1995). (C and D) qRT-PCR comparing the effects of Pax6 absence (C) or conditional loss (D) on the cortical expression of a set of genes found to have altered expression in our microarray analysis. All differences are significant (means ± SEM; Student’s t test, ^∗^p < 0.05, n > 3 embryos per genotype in all cases). (E) qRT-PCR comparing the mean levels of *Pax6* and *Cdk6* (±SEM; n = 4 embryos of each genotype) in the rostral and caudal thirds of E12.5 cortex from WT and *PAX77* (Pax6 overexpressing) embryos. *Pax6* rostrally: ^∗^p < 0.001, caudally ^∗^p < 0.0001; *Cdk6*: ^∗^p < 0.0016 (Sidak’s multiple-comparisons test). See also Figures S4, S5, and Table S1.

We used Gene Ontology (GO) databases to identify the biological processes most frequently associated with the sets of up- and downregulated transcripts. The ten most significantly overrepresented processes for each set are given in Figure 3A. Notably, in the context of the present work, the terms “cell cycle,” “cell division,” “mitosis,” and “DNA replication” appear in the list of processes associated with the upregulated transcripts but not among the list of processes associated with the downregulated transcripts. This agrees with our overall hypothesis that Pax6 is normally a net repressor of the transcription of genes associated with cell proliferation. The regulated genes that are most closely associated with the cell cycle are listed in Figure 3B along with their most-relevant known functions.

We selected a subset of cell-cycle and other genes regulated by Pax6 in our microarrays and compared their lateral cortical expression levels between E12.5 *Pax6*^*+/+*^ and *Pax6*^*−/−*^ embryos by qRT-PCR (Figure 3C). All showed significant differences. We used qRT-PCR to compare the expression of *Cdk6*, *Ccnd1* (*Cyclin D1*), *Cdca7*, and *Smad2* in control and iKO (*Pax6*^*loxP*/*loxP*^; *Emx1-CreER*^T2^) cortex 72 hr after tamoxifen administration at E13.5, and, as in the *Pax6*^*−/−*^ cortex, we found significant increases in the levels of all four in the iKO cortex (Figure 3D). Data on the patterns of expression of these four genes shown by in situ hybridization are included in Figures S5E–S5L.

In the light of our discoveries from these experiments (as described in the following sections), we carried out an additional experiment to test whether the levels of expression of *Cdk6* were affected by controlled overexpression of Pax6, as occurs in the *PAX77* line (Manuel et al., 2007). Pax6 protein levels in these mice are elevated ∼2-fold, although the pattern of expression remains normal (Manuel et al., 2007). The levels of *Pax6* and *Cdk6*, as measured by qRT-PCR, in samples of rostral and caudal cortex varied significantly with both area and genotype (two-way ANOVAs; Figure 3E). In WTs, *Pax6* and *Cdk6* were correlated inversely: *Pax6* levels were significantly higher and *Cdk6* levels were significantly lower in rostral than in caudal cortex (p < 0.0001 and p < 0.0009, respectively; Figure 3E, black lines). In *PAX77* embryos, *Pax6* levels were elevated both rostrally and caudally and there was a significant reduction of *Cdk6* levels caudally. Whereas elevated Pax6 levels repressed *Cdk6* expression caudally, repression was not detected rostrally. It is possible that rostral increases might have little effect on *Cdk6* expression if Pax6-mediated repression of *Cdk6* in WTs is already relatively close to maximum in this region.

### Predicted Pax6-Binding Sites around the *Cdk6* Gene

As a next step toward defining a biochemical pathway through which Pax6 might regulate cortical progenitor cell cycles, we used bioinformatics to identify potential Pax6 binding sites in genomic regions surrounding some of the cell-cycle genes regulated by Pax6. Using a 21 bp consensus binding motif that is known from previous work to be recognized by the Pax6 paired domain (P6CON; Epstein et al., 1994a, 1994b), we identified a particularly large number of putative Pax6 binding sites within 10 kb on either side of the *Cdk6* coding sequence and focused further work on testing for direct regulation of this gene by Pax6.

We used two position weight matrix (PWM) databases, TRANSFAC and JASPAR, and identified 14 putative Pax6 binding sites within the 10 kb genomic regions immediately upstream and downstream of the *Cdk6* coding region. These candidate sites were filtered by conservation analysis across five vertebrate species (mouse, rat, dog, chimpanzee, and human) using the Mulan program. Five putative binding sites (BS1–BS5; Figure S6A) with at least 85% similarity to P6CON and >90% identity across the five vertebrate species were defined for experimental testing. The murine *Cdk6* promoter has not been characterized, but the human *CDK6* promoter has (Cram et al., 2001). By aligning its sequence against the mouse genome (NCBI Build 37, UCSC mm9) we identified a 2.3 kb region flanking the 5′ end of the murine *Cdk6* gene with ∼80% homology to the human *CDK6* promoter sequence. This region, which probably contains the murine *Cdk6* promoter, also contains one of the five putative Pax6 binding sites (BS1; Figure 5C).

### Evidence for Pax6 Binding Sites around the *Cdk6* Gene from Electrophoretic Mobility Shift Assays

**Figure 4:**
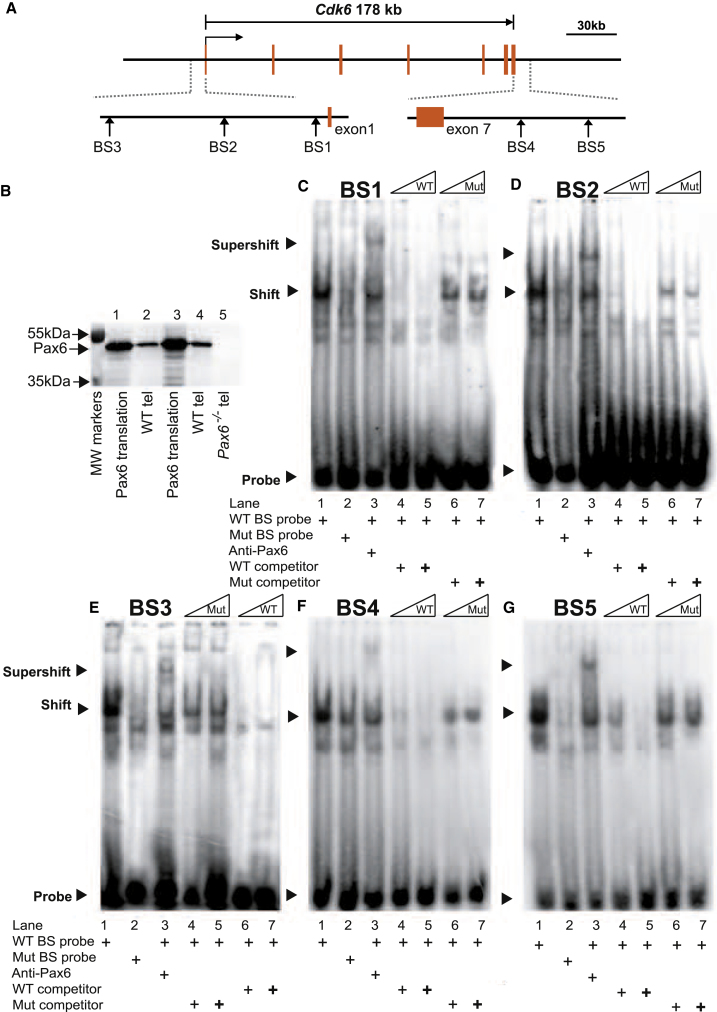
EMSAs Show that Pax6 Can Bind to Predicted Binding Sites (BS1–BS5) at the *Cdk6* Locus (A) Map of the five binding sites (BS1–BS5) at the *Cdk6* locus (sequences in Figure S6A). (B) Western blot analysis of Pax6 protein expressed by the TNT In Vitro Transcription/Translation System. Lanes were loaded with 2 μl (lane 1) and 4 μl (lane 3) of in vitro-translated Pax6 protein, 20 μg (lane 2) and 40 μg (lane 4) of *Pax6*^+/+^ telencephalic protein, or 40 μg of *Pax6*^*−/−*^ telencephalic protein (lane 5). (C–G) EMSAs. Arrowheads indicate free probe (Probe) at the bottom of each gel (probe is WT in lanes 1 and 3–7, and mutant (Mut) in lane 2), probe-protein complexes (Shift), and probe-protein-antibody complexes (Supershift). In all cases, a specific gel shift by binding of Pax6 to potential Pax6 binding sites was clearly detected (lane 1) and was greatly reduced or abolished by mutation of the Pax6 binding sites (lane 2). Binding specificity was confirmed by preincubation with anti-Pax6 antibody, resulting in supershifted complexes (lane 3). Visible shifts were diminished or abolished (in a dose-dependent manner in most cases) by addition of excess nonradiolabeled WT probes to compete with radiolabeled probes, whereas excess amounts of nonradiolabeled mutant probes competed much less effectively (lanes 4–7). See also Figure S6A.

We tested whether BS1–BS5 (Figure 4A) could specifically bind Pax6, using Pax6 protein generated by in vitro translation (Figure 4B). Electrophoretic mobility shift assays (EMSAs) are shown in Figures 4C–4G. The migration of radioactively labeled oligonucleotides containing each predicted Pax6 binding site (BS1–BS5) was retarded by binding to Pax6 protein (shift) and retarded further upon addition of an anti-Pax6 antibody (supershift). Radiolabeled oligonucleotides containing mutations that abolish the Pax6 consensus binding site in BS1–BS5 (Figure S6A) showed reduced or no retardation (compare lanes 1 and 2). When excess unlabeled WT oligonucleotides were added as competitors, they weakened or abolished the shifted bands in all cases. In contrast, unlabeled mutant oligonucleotides (Figure S6A) were unable to compete effectively with the labeled WT oligonucleotides. These assays demonstrate that Pax6 protein can bind specifically to sequences representing the predicted Pax6 binding sites BS1–BS5.

### Pax6 Binds to Sites around *Cdk6* In Vivo

**Figure 5:**
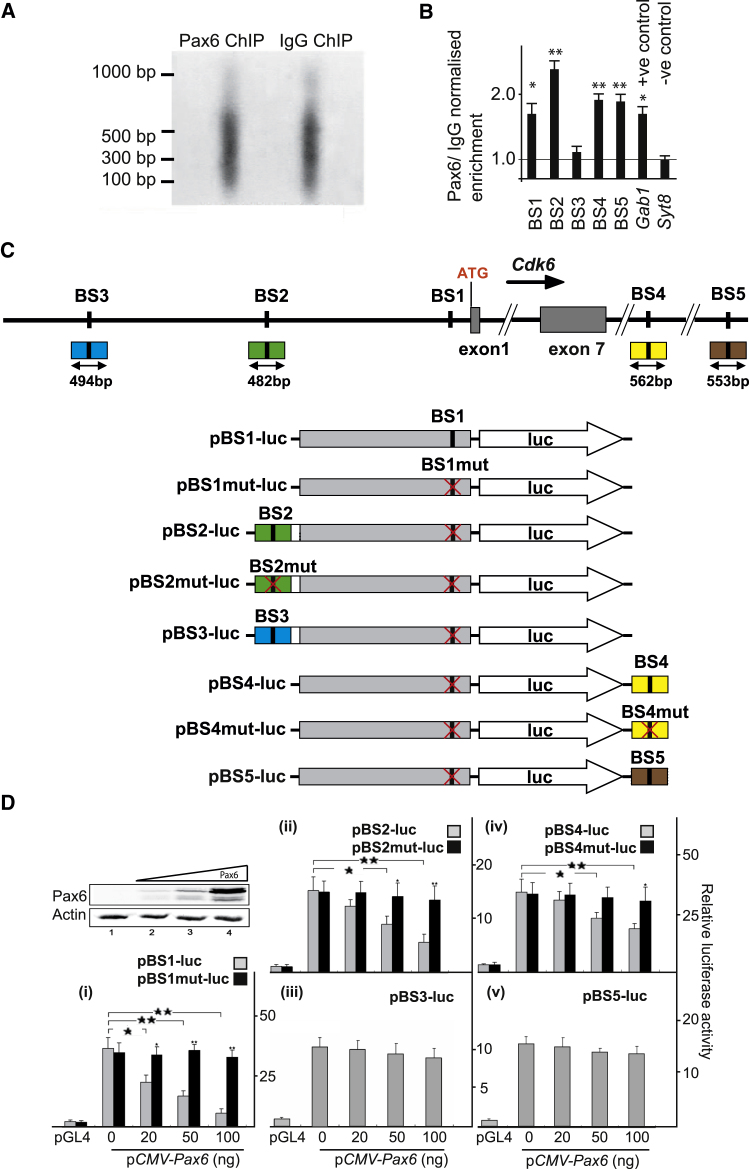
Evidence that Sites around the *Cdk6* Gene Bind Pax6 In Vivo and Repress Gene Expression In Vitro (A) Sonication of cortical chromatin gave mainly 100–600 bp genomic fragments for ChIP with antibodies against either Pax6 (experiment) or IgG (control). (B) Results of qChIP on DNA from E12.5 dorsal telencephalon using anti-Pax6 and anti-IgG antibodies followed by qPCR to test for enrichment of each putative Pax6 binding site. For each binding site, the amount of qPCR product obtained with anti-Pax6 antibody was first expressed relative to that obtained with anti-IgG antibody. The resulting ratio was expressed relative to the average ratio obtained with primers for a sequence from the *Syt8* gene that does not bind Pax6 (which was set to 1.0; Sansom et al., 2009). A *Gab1* sequence known to bind Pax6 (Sansom et al., 2009) was used as a positive control. Means ± SEM (n = 3); ^∗^p *<* 0.05 and *^∗∗^*p *<* 0.01 (Student’s t test). (C) Constructs made to test BS1–BS3 located 5′ to exon 1, and BS4 and BS5 located 3′ to exon 7. The position of each binding site is indicated in the schematic drawing, and the size and position of the fragments that were used to generate the constructs are indicated directly beneath. Black vertical bars in the plasmid schematics denote WT binding sites and mutant sites are indicated by red crosses. (D) Results obtained using these constructs to measure firefly luciferase activity (relative to a *Renilla* luciferase control) in HEK293 cells made to express a range of levels of Pax6 using a CMV-*Pax6* construct added at 0, 20, 50, or 100 ng per transfection (the western blot shows Pax6 expression resulting from increasing doses, with actin levels monitored as a loading control). Data labeled pGL4 are from the promoterless vector used to make the constructs with no added inserts as negative controls. All eight constructs induced significant luciferase activity in the absence of Pax6. Data are from three independent experiments. Means ± SEM (n = 3); ^∗^p *<* 0.05 and *^∗∗^*p *<* 0.01 (Sidak’s multiple-comparisons test). See also Figure S6B and Tables S2 and S3.

We extracted chromatin from E12.5 cortex to test for binding of Pax6 to the predicted Pax6 binding sites BS1–BS5 in vivo by quantitative chromatin immunoprecipitation (qChIP; Figures 5A and 5B). Primer pairs were selected to measure, by qPCR, the relative levels of short fragments spanning each predicted binding site (Figure S6B). Primers for sequences from the genomic regions of *Gab1* and *Syt8* that were previously shown to be Pax6 bound and Pax6 nonbound, respectively, were used to generate positive and negative control data (Sansom et al., 2009). Following the qPCR, values for Pax6/immunoglobulin G (IgG) normalized enrichment were expressed relative to the average value for *Syt8* (Figure 5B). DNA sequences that included four of the five *Cdk6* Pax6 predicted binding sites (BS1, BS2, BS4, and BS5) were significantly enriched by amounts similar to or greater than that of the *Gab1* positive control (Figure 5B). There was no evidence for enrichment of the BS3 sequence. Taken together, the EMSA and ChIP results indicate that Pax6 has the potential to bind all five *Cdk6* sites (BS1–BS5), but binds to only four of them in E12.5 cortex in vivo.

### Pax6 Directly Represses Cdk6 Expression

We next examined the functionality of each of the Pax6 binding sites (BS1–BS5) using luciferase assays in cells that do not express endogenous PAX6 (HEK293 cells). We generated a set of eight luciferase reporter constructs to test each site individually (Figure 5C). We first cloned a 2.3 kb upstream fragment encompassing the putative *Cdk6* promoter and containing only BS1 into the promoterless luciferase reporter plasmid pGL4.10 to generate the plasmid pBS1-luc. This produced a substantial increase in relative luciferase activity compared with cells transfected with pGL4 vector alone (Figure 5Di). Cotransfection of increasing amounts of the Pax6 expression construct p*CMV-Pax6* (Figure 5D) led to a dose-dependent reduction in relative luciferase activity (Figure 5Di). To test whether this reduction was due to Pax6 binding to BS1, we mutated BS1 exactly as done for the EMSAs (Figure S6A) to generate pBS1mut-luc (Figure 5C). The mutation abolished Pax6-dependent suppression of luciferase activity (Figure 5Di), indicating that binding of Pax6 to site BS1 can repress transcription from the *Cdk6* promoter.

We then evaluated each of the four remaining Pax6 binding sites (BS2–BS5) individually. Short DNA fragments spanning each of the binding sites were cloned into plasmid pBS1mut-luc (which drives reporter expression and is not itself repressed by Pax6). PCR fragments including BS2 or BS3 were placed immediately upstream of the 2.3 kb promoter in pBS1mut-luc, whereas fragments including BS4 or BS5 were placed downstream of the luciferase coding sequence, mimicking their normal position relative to the *Cdk6* transcription start site (Figure S6A). Constructs that contained sites BS2 or BS4 both showed a clear dose-dependent decrease in luciferase activity in response to Pax6, which was abolished when either BS2 or BS4 was mutated (Figures 5Dii and 5Div), indicating that binding of Pax6 to either of these sites can repress transcription. In contrast, constructs containing BS3 or BS5 showed no decrease in luciferase activity in response to Pax6 (Figures 5Diii and 5Dv), suggesting that neither BS3 (which did not bind Pax6 in vivo; Figure 5B) nor BS5 mediates repression by Pax6, at least in the present context. Together, these results indicate that at least three Pax6 binding sites around *Cdk6* (BS1, BS2, and BS4, all three of which bind Pax6 in vivo; Figure 5B) can mediate Pax6-dependent suppression of transcription.

### Pax6 Regulates the Phosphorylation of pRb

Previous work has shown that Cdks promote progression through the cell cycle (Malumbres and Barbacid, 2005). Of particular relevance to the present work, a previous in vitro study showed that a dominant-negative *Cdk6* construct inhibited E12.5 cortical progenitor proliferation (Ferguson et al., 2000). We observed a similar effect in vivo following cortical electroporation of the same construct (Figures S7A–S7E). We also observed reduced apical progenitor cell division in E12.5 *Cdk6*^*−/−*^ embryos (Malumbres et al., 2004) compared with controls (Figures S7F–S7H), consistent with a normal role for Cdk6 in regulating cortical progenitor proliferation in vivo. Cyclin/Cdk complexes induce hyperphosphorylation of pRb. This hyperphosphorylation antagonizes the ability of pRb to bind and sequester transcription factors of the E2F family, and free E2F proteins promote transition through the cell cycle (Polager and Ginsberg, 2008). We predicted, therefore, that increased phosphorylation of pRb might provide a link between the upregulation of Cdk6 and the increased proliferation of cortical progenitors that occurs in the absence of Pax6.

**Figure 6:**
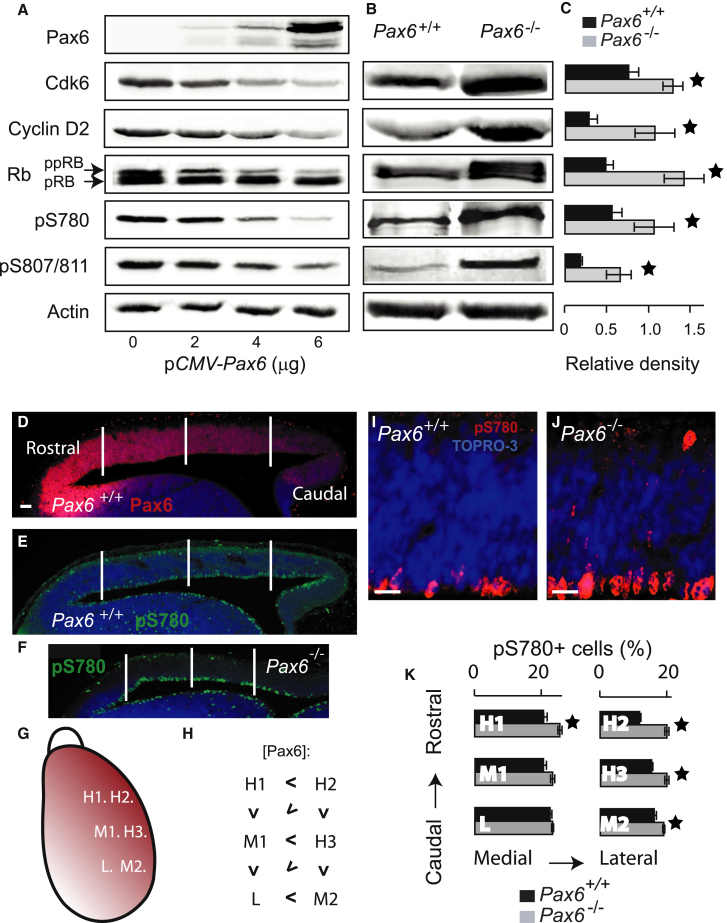
Pax6 Suppresses Hyperphosphorylation of pRb (A) Western blots showing that transient transfection of HEK293 cells with increasing amounts of Pax6 expression plasmid *pCMV-Pax6* resulted in increasing levels of Pax6 and decreasing levels of Cdk6, cyclin D2, the hyperphosphorylated form of pRb (ppRB), pRb phosphorylated at serine 780 (pS780), and pRb phosphorylated at serine 807/811 (pS807/811). Similar results were obtained in three independent experiments. (B) Western blots on protein extracted from E12.5 cortices showing increased levels of Cdk6, cyclin D2, ppRb, pS780, and pS807/811 in the absence of Pax6. (C) Densitometry on bands representing Cdk6, cyclin D2, pRb, pS780, and pS807/811 from western blots such as those in (B). For each protein extract, values were normalized to the β-actin level in that sample. Means ± SEM; *^∗^*p < 0.05, Student’s t test; n = 3 independent experiments in each case. (D–F) Immunohistochemistry showing expression of pS780 and Pax6 in parasagittal sections of E12.5 *Pax6*^*+/+*^ and *Pax6*^*−/−*^ cortex. Vertical lines mark the rostrocaudal levels of the quantifications in (G), (H), and (K); counts were in 150 μm × 30 μm areas oriented along the ventricular surface. Scale bar, 100 μm. (G–K) Quantification of the percentages of pS780+ cells in three cortical areas expressing relatively high (H1–H3), medium (M1 and M2), and low (L) levels of Pax6 in two rostral-to-caudal rows of sampling areas (one row medial to the other, laid out as illustrated on a dorsal view of the right hemisphere in (G). The relationships between the relative levels of Pax6 in each position [<] are summarized in (H). (I and J) Rostrolateral sections of cortex from E12.5 *Pax6*^*+/+*^ and *Pax6*^*−/−*^ embryos immunoreacted for pS780. Scale bars, 25 μm. (K) Means ± SEM; *^∗^*p < 0.05, Student’s t test; n = 9 embryos of each genotype. See also Figure S7.

We first tested the effects on pRb phosphorylation of transfecting Pax6 nonexpressing cells (HEK293) with increasing amounts of the Pax6 expression construct p*CMV-Pax6*. Western blots showed an inverse relationship between Pax6 and Cdk6/cyclin D2 (*Ccnd2*) protein levels (Figure 6A), consistent with our finding that Pax6 represses the expression of both genes (Figure 3B). Loss of cyclin/Cdk was associated with loss of the hyperphosphorylated form of pRb (ppRb; Figure 6A). Because cyclin/Cdk complexes are known to phosphorylate pRb at specific residues, including Ser-780 and Ser-807/811 (Kitagawa et al., 1996; Zarkowska and Mittnacht, 1997; Ely et al., 2005), we used antibodies that recognize pRb that is phosphorylated specifically at these positions (pS780 and pS807/811). The levels of both phosphorylated forms declined with increasing Pax6 levels (Figure 6A). We then used western blots to compare the levels of these proteins and phosphorylated forms of pRb in E12.5 WT and *Pax6*^*−/−*^ cortex (Figure 6B; see quantifications of three repeats in Figure 6C). In all cases, the levels were significantly increased in mutants.

We also examined the distribution of pRb phosphorylated at Ser-780 in the E12.5 cortex of WT and *Pax6*^*−/−*^ embryos by immunohistochemistry (Figures 6D–6K). Most pS780-positive cells were located along the ventricular edge, where progenitor cells undergo M phase and enter the G1 phase of the cell cycle. In WT embryos, staining for pS780 appeared more intense in the caudal cortex, where Pax6 levels are relatively low (Figures 6D and 6E). In *Pax6*^*−/−*^ cortex, the intensity of pS780 staining appeared to be increased particularly in the rostral cortex (Figure 6F). The proportions of cells that were pS780-positive were counted in regions of cortex that normally express different relative levels of Pax6 (Figures 6D–6K). In WT cortex, the proportions of pS780-positive cells were lowest in the rostrolateral (i.e., [Pax6]^high^) cortex (Figure 6K). In *Pax6*^*−/−*^ cortex, there were significant increases in the proportions of pS780-positive cells in regions that would normally express the highest levels of Pax6 (i.e., rostral and lateral, labeled H1–H3 and M2 in Figure 6K), but not in regions that would normally express lower levels of Pax6. These changes resulted in an abolition of normal regional differences in the proportions of pS780-positive cells, providing further evidence that high levels of Pax6 normally suppress cyclin/Cdk-mediated pRb phosphorylation in cortical progenitors in vivo.

## Discussion

**Figure 7:**
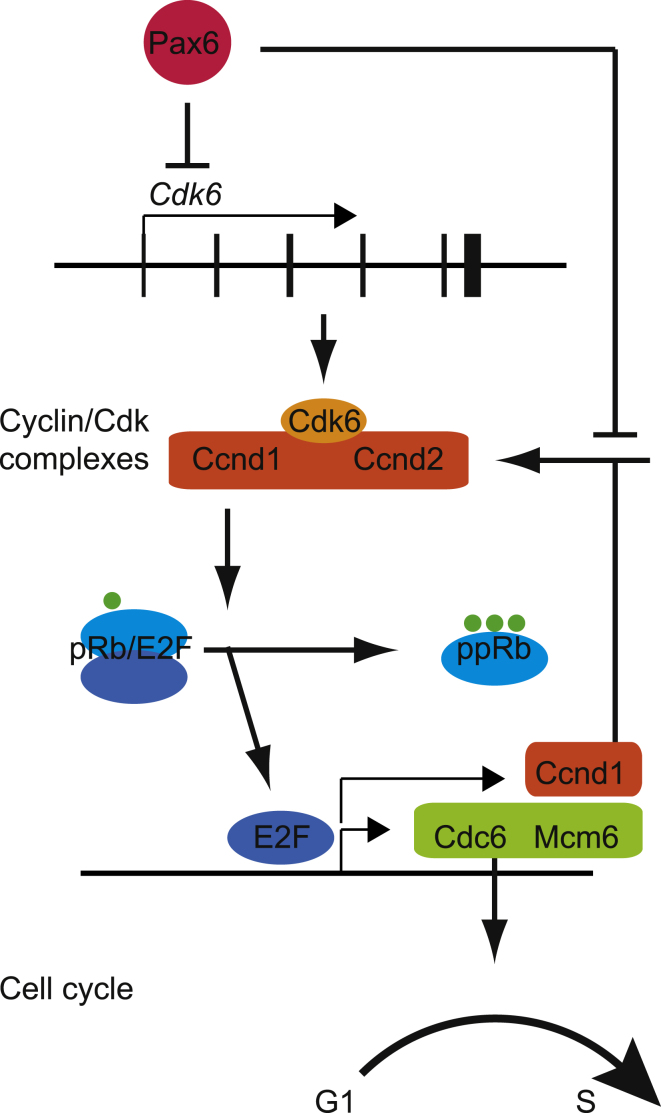
A Pathway by which Pax6 Regulates Cortical Progenitor Cell Cycles In this model, Pax6 directly represses *Cdk6* expression and, either directly or indirectly, the expression of other cyclins (Ccnds)/Cdks. This limits the hyperphosphorylation of pRb, which is catalyzed by cyclin/Cdk complexes. This limits the release of E2F transcription factors, which bind to pRb. This limits the expression of genes such as *Cdc6* and *Mcm6*, which promote G1 to S phase transition, and *Ccnd1* (*Cyclin D1*), thereby dampening a positive feedback loop that would promote proliferation.

Our findings allow us to propose a model of one relatively direct route through which Pax6 can influence cortical progenitor Tcs (Figure 7). In summary, our results indicate that by repressing *Cdk6* (through binding to sites close to the *Cdk6* coding sequence) and *Cyclin D1/2* (either directly or indirectly), Pax6 can limit the levels of cyclin/Cdk complexes and hence the phosphorylation of pRb, one of the primary substrates of Cdks in G1 phase progression (Ferguson and Slack, 2001). Limiting the phosphorylation of pRb suppresses the release from pRb/E2F complexes of E2F transcription factors, which promote G1/S transition and hence proliferation (Harbour et al., 1999). E2F’s direct targets include *Cdc6*, *Mcm6*, and *Cdca7* (Di Stefano et al., 2003; Lee et al., 2000; Polager and Ginsberg, 2008; Goto et al., 2006), and, in agreement with our model, we identified all three as being upregulated in *Pax6*^*−/−*^ cortical progenitors. Cdc6 and Mcm6 are involved in the onset of S phase by regulating DNA replication, and one of their main functions is to unwind DNA for replication (Bochman and Schwacha, 2009; Knockleby and Lee, 2010). The functions of Cdca7 are currently unclear. Also included in our model is a feedback loop involving cyclin D1 (*Ccnd1*), which is known to be directly and positively regulated by E2Fs (Di Stefano et al., 2003; Lee et al., 2000; Polager and Ginsberg, 2008). By limiting E2F-mediated cyclin D1 expression, Pax6 can limit the influence of this positive feedback. If Pax6 is deleted, this positive feedback loop will be enhanced, providing a drive for cell-cycle progression.

These new findings provide an important framework for future work. The results of previous studies left the issue of whether Pax6 directly regulates the transcription of cell-cycle genes in the cortex highly uncertain. Both previous work and the present screen have shown that Pax6 can regulate, in some cases directly, the transcription of many other transcription factor genes, such as *Ngn2* and *Ascl1*, that themselves regulate cell proliferation (Scardigli et al., 2003; Holm et al., 2007; Tuoc and Stoykova, 2008; Sansom et al., 2009; Castro and Guillemot, 2011; Castro et al., 2011). There was, therefore, a strong possibility that Pax6 might act on the cell cycle only indirectly by controlling the expression of other transcription factors (Sansom et al., 2009). Here we show evidence for a direct mechanism, but most likely Pax6’s control of the cell cycle in cortical progenitors is mediated by both direct and indirect mechanisms. It seems extremely unlikely that Pax6’s direct actions on the cell cycle are mediated exclusively through repression of *Cdk6*. We view our model as a start toward building an ultimately much more complex understanding of a doubtlessly large network of interactions between numerous directly and indirectly regulated molecular pathways that mediate Pax6’s actions on cortical progenitor cell cycles.

The challenges involved in identifying functionally important transcription factor binding sites that regulate a specific target gene are well known (e.g., see a recent review by Biggin, 2011). Our experiments using EMSAs showed that five predicted sites around *Cdk6* can bind Pax6, ChIP showed that four of them bind Pax6 in cortical progenitors in vivo, and luciferase assays showed that three of these four sites (one of which is within the likely *Cdk6* promoter region) respond to Pax6 by repressing gene expression. The failure to detect Pax6 binding to BS3 by ChIP suggests low or no occupancy of this relatively distant site by Pax6 in cortical progenitors in vivo, in line with previous work indicating that many potential transcription factor binding sites are unoccupied in vivo (Carr and Biggin, 1999; Biggin, 2011). The finding that BS3 and BS5 did not mediate suppression might indicate that these sites do not mediate Pax6 regulation of *Cdk6* even if they bind Pax6, but could be explained in other ways. For example, the function of some sites might depend on simultaneous binding at a particular combination of sites. Overall, therefore, we draw a strong conclusion from our evidence for binding and functional repression of BS1, BS2, and BS4 irrespective of the currently unclear nature of the interaction between Pax6 and BS3 and BS5.

Our in vivo results indicate that at the onset of corticogenesis, the repressive effect of Pax6 on progenitor cell proliferation and the phosphorylation of pRb is localized to regions of cortex where Pax6 expression is normally highest, since loss of Pax6 either constitutively or conditionally had the greatest effects in those areas. As corticogenesis proceeds, Pax6 expression becomes more uniform across the cortex and the effects of Pax6 loss become more widespread, indicating a relationship between the levels of expression and the proliferative effect. Further evidence for a relationship between cortical Pax6 levels and progenitor proliferation comes from previous studies in which Pax6 overexpression was shown to decrease progenitor proliferation rates (Manuel et al., 2007; Georgala et al., 2011a). This effect, as might be anticipated, is the opposite of what we observed to result from the loss of Pax6. In agreement with our model, we have shown here that Pax6 overexpression can repress *Cdk6* levels.

The early regional effects of Pax6 on proliferation are important in the context of understanding how the cerebral cortex becomes divided into regions with specific cytoarchitectures and functions. The early embryonic cortex is patterned by concentration gradients of several high-level transcription factors, including Pax6, but the mechanism by which the Pax6 gradient might contribute to the specification of cortical areas remains unclear (Bishop et al., 2000; Manuel et al., 2007). By affecting cell-cycle parameters in a region-specific manner, Pax6 can regulate regional differences in two critical aspects of cortical neuronal generation, namely, the numbers of neurons that are produced and their fates, both of which are likely to influence cytoarchitecture and function. There is now good evidence that cortical cell fates depend at least in part on the length of the cell cycle, in particular its G1 phase, which is a period of increased sensitivity to differentiation signals (Dehay and Kennedy, 2007; Pilaz et al., 2009). It is likely, therefore, that Pax6 can contribute to regional differences across the early developing cortex because of its graded expression levels combined with its ability to influence directly and in a concentration-dependent manner the levels and hence the functions of cell-cycle proteins such as Cdks and cyclins.

## Experimental Procedures

### Mice

Mice were bred in accordance with the guidelines of the UK Animals (Scientific Procedures) Act 1986. For constitutive inactivation of *Pax6*, we used the *Pax6*^*Sey*^ allele (designated as *Pax6*^*−*^ here; Hill et al., 1991). For controlled overexpression of Pax6, we used the *PAX77* transgenic line (Manuel et al., 2007). For conditional inactivation of *Pax6*, we used *Pax6*^*loxP*^ (Simpson et al., 2009), BAC transgenic strain *Emx1-CreER*^T2^ (Kessaris et al., 2006), and *R26R-YFP* (Srinivas et al., 2001) alleles. Cre expression was induced with 10 mg (at E10.5) or 12.5 mg (at E13.5) tamoxifen (orally, 50 mg ml^−1^; Sigma). To separate *Pax6*-expressing cells for gene profiling, we used the *DTy54* transgene (Tyas et al., 2006). Electroporations were carried out as described previously (Saito, 2006) using either a control GFP-expressing construct or a dominant-negative *Cdk6* (*DN-Cdk6*) and GFP-expressing construct described previously (Ferguson et al., 2000).

### Immunohistochemistry

IdU and BrdU were used to label proliferating cortical cells as described in Martynoga et al. (2005). The primary antibodies used were mouse anti-BrdU/IdU (1:50–1:100; BD Biosciences), rat anti-BrdU (ab6326, 1:50; Abcam), mouse anti-phosphorylated histone H3 (ab1791, 1:200; Abcam), mouse anti-Pax6 (1:200; DSHB), rabbit anti-GFP/YFP (ab290, 1:500; Abcam), and anti-pRb pS780 (1:200; Cell Signaling). Nuclei were counterstained with TOPRO-3 (1:1,000; Molecular Probes).

### RNA Analyses

Telencephalic cells were dissociated with papain (20 U ml^−1^; Biochemical Dissociation Kit; Worthington) and GFP-expressing cells were sorted by FACS. RNA was extracted from dissociated cells or from telencephalic tissue using QIAshredder Spin Columns and QIAGEN RNeasy Kits; any traces of genomic DNA were removed by on-column DNase digestion. For microarray experiments, the quality of each sample was assessed using the Agilent Bioanalyzer to obtain an RIN of 1–10 (10 = highest-quality intact RNA). The Agilent Low RNA Input Linear Amplification Kit was then used to produce complementary RNA (cRNA) labeled with either Cyanine 3 (Cy3) or Cyanine 5 (Cy5) fluorescent labels. Labeled cRNA samples were hybridized to Agilent Dual-dye Whole Mouse Genome Arrays (G4122A). After hybridization, arrays were scanned in an Agilent Scanner for Cy3 and Cy5, and images were analyzed using Agilent’s Feature Extraction Software (version 7.1). Normalization and statistical analysis of the microarray data were performed using R-based software (http://www.R-project.org). For qRT-PCR, reverse transcription and real-time amplification were carried out using standard protocols with the primers listed in Table S1. All mRNA levels were expressed relative to those for glyceraldehyde 3-phosphate dehydrogenase (GAPDH). In situ hybridizations were carried out using standard protocols.

### Bioinformatics

GO analysis was carried out with WebGestalt (WEB-based GEne SeT AnaLysis Toolkit; http://bioinfo.vanderbilt.edu/webgestalt/) and FunNet Transcriptional Networks Analysis (http://www.funnet.info), using the GO (http://www.geneontology.org) and Kyoto Encyclopedia of Genes and Genomes (KEGG; http://www.genome.jp/kegg) databases. To predict Pax6-binding sites, we used TRANSFAC (professional 2009.1) and JASPAR (http://jaspar.cgb.ki.se) combined with conservation analysis using the Mulan (http://mulan.dcode.org) program. To reduce false positives, the threshold scores of the core motif match and the matrix match were set to 0.8 in the TRANSFAC program. The scanning cutoff was chosen such that the probability of getting a false-positive prediction in sequences of 500 bp length was <5% (fixed type I error). The probability of a given DNA sequence functioning as a *cis*-regulatory element was set to 80.0% for the JASPAR database.

### EMSAs

Pax6 proteins were obtained from full-length *Pax6* cDNA using the TNT Quick Coupled Transcription/Translation System (Promega). Double-stranded oligonucleotide probes containing WT or mutant Pax6 binding sites (Figure S6A) were radiolabeled. Pax6 polyclonal rabbit antibody (Covance) was used for supershifts. Competition assays used a 50- to 100-fold molar excess of competitor probes in each binding reaction.

### qChIP

Chromatin was extracted from 20 E12.5 mouse cortices. DNA-protein complexes were precipitated with anti-Pax6 antibody (Covance) or with anti-igG antibody (Abcam). ChIP was performed as described by Sansom et al. (2009). Primer pairs were selected to measure, by qPCR, the relative levels of fragments, each of which included one of the predicted binding sites (Figure S6B).

### Luciferase Assays

Fragments around *Cdk6* were isolated from a BAC clone (RP23-53B17) or genomic DNA using the primers listed in Table S2 and cloned into the pGL4.10 promoterless *firefly* luciferase reporter vector (Promega). Site-directed mutagenesis used the QuikChange Site-Directed Mutagenesis Kit (Stratagene) with the PCR primers listed in Table S3. HEK293 cells were transfected using Lipofectamine 2000 (Invitrogen). Pax6 was expressed using the p*CMV-Pax6* construct, generated by inserting full-length Pax6 cDNA into p*CMV-Script* plasmid (Stratagene). The *Renilla* luciferase vector was p*RLSV40* (Promega). HEK293 cells were harvested 48 hr after transfections and analyzed with the Dual Luciferase Reporter Assay System (Promega).

### Western Blots

The primary antibodies were mouse anti-Pax6 (1:200; DSHB), rabbit anti-Pax6 (1:500; Covance), rabbit anti-Cdk6 (1:500; Santa Cruz), rabbit anti-cyclin D2 (1:200; Santa Cruz), mouse anti-pRb (1:200; BD PharMingen), rabbit anti-phospho-pRb (Ser780, 1:1,000; Cell Signaling), rabbit anti-phospho-pRb (Ser807/811, 1:1,000; Cell Signaling), and rabbit anti-beta actin (1:2,000; Abcam). Alexa-coupled secondary antibodies were used and blots were quantified using the Li-Cor scanning system.

## Accession Numbers

The GEO accession number for the microarray data reported in this paper is GSE38703.
