# Supplementary material for: Pax6 Exerts Regional Control of Cortical Progenitor Proliferation via Direct Repression of Cdk6 and Hypophosphorylation of pRb
Source: Neuron. 2013 Apr 24;78(2):269–84. doi: 10.1016/j.neuron.2013.02.012 (PMC3898967; doi:10.1016/j.neuron.2013.02.012)
Supplement: Document S1. Figures S1–S7 and Tables S1–S3 [file mmc1.pdf]

Neuron, Volume *78*
Supplemental Information

Pax6 Exerts Regional Control of Cortical Progenitor Proliferation via Direct Repression of *Cdk6* and Hypophosphorylation of pRb

Da Mi, Catherine B. Carr, Petrina A. Georgala, Yu-Ting Huang, Martine N. Manuel, Emily Jeanes, Emi Niisato, Stephen N. Sansom, Frederick J. Livesey, Thomas Theil, Kerstin Hasenpusch-Theil, T. Ian Simpson, John O. Mason, and David J. Price

SUPPLEMENTAL INVENTORY

Figure S1. Proliferation defects were not detected in embryonic *Pax6*$^{+/-}$ cortex.

Associated with Fig. 1.

Figure S2. Timing of Pax6 deletion from iKO embryos. Associated with Fig. 2.

Figure S3. Counts of apical mitoses in conditional mutants. Associated with Fig. 2.

Figure S4. Separation of *Pax6*-expressing cells from *Pax6*$^{+/+}$ and *Pax6*$^{-/-}$ cortex using FACS. Associated with Fig. 3.

Figure S5. *In situ* hybridisation experiments. Associated with Fig. 3.

Figure S6. Details of five predicted Pax6 binding sites (BS1-5) around the *Cdk6* coding sequence and potential binding sites tested and primers used in ChIP experiments. Associated with Figs. 4 and 5.

Figure S7. Inhibition or loss of Cdk6 slows cortical progenitor proliferation *in vivo*. Associated with Fig. 6.

Table S1. Primers for qRT-PCR. Associated with Fig. 3.

Table S2. PCR primers for cloning. Associated with Fig. 5.

Table S3. PCR primers for mutagenesis. Associated with Fig. 5.

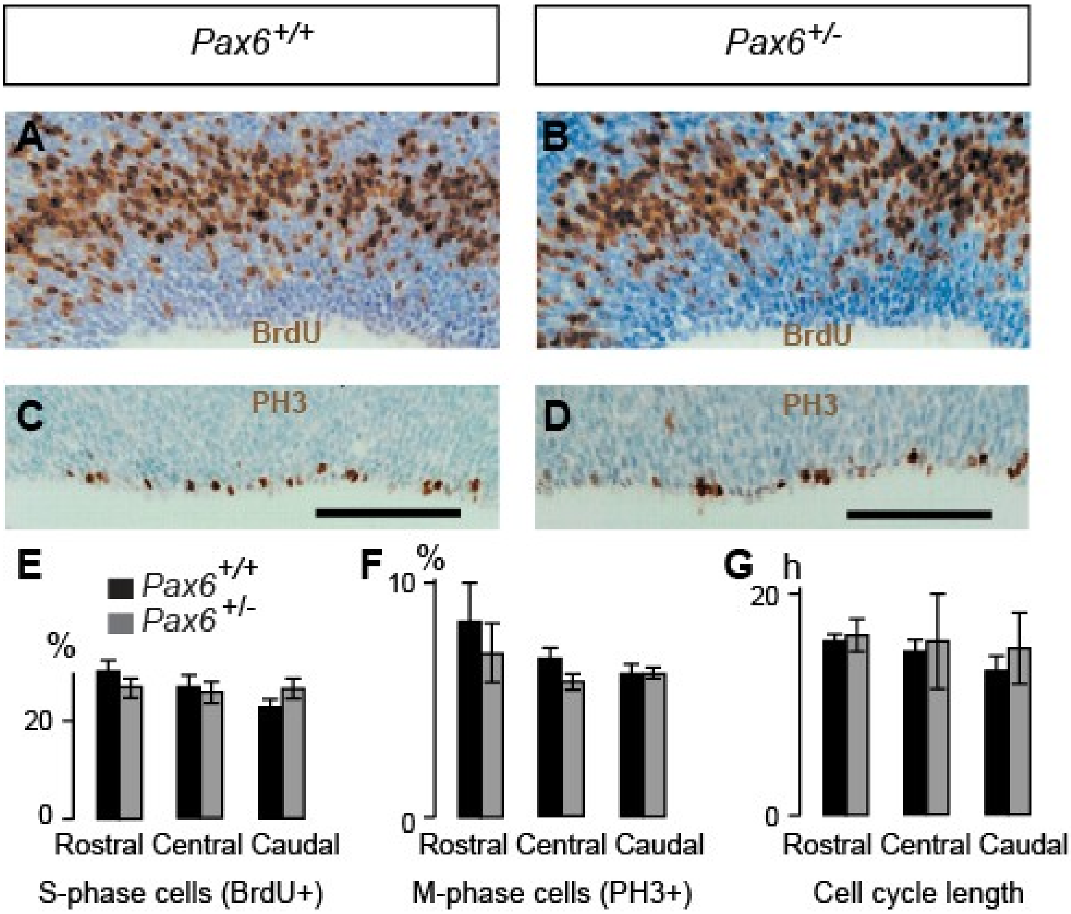

Figure S1. Proliferation defects were not detected in embryonic *Pax6*$^{+/-}$ cortex (related to Fig. 1)**.** (A-D) Immunohistochemistry for (A,B) BrdU on tissue fixed 30 minutes after BrdU administration to pregnant dams and (C,D) phosphorylated histone 3 (PH3). Sections were counterstained with cresyl violet; scale bar = 100µm. (E) Proportions of all ventricular zone cells that were BrdU-labelled were counted in 100µm-wide bins. (F) Proportions of all cells along the ventricular surface that were PH3-labelled were counted in 100µm-wide strips. (G) Cell cycle lengths were estimated using methods summarized in Fig. 1 of this paper and described in Martynoga et al. (2005). Counts were in rostral, central and caudal cortical areas (high, medium and low Pax6 expression, respectively). All values are means ± standard errors of the means (n = 3 animals in each case). There were no statistically significant differences between genotypes (Student's t-tests, p>0.05 in all cases).

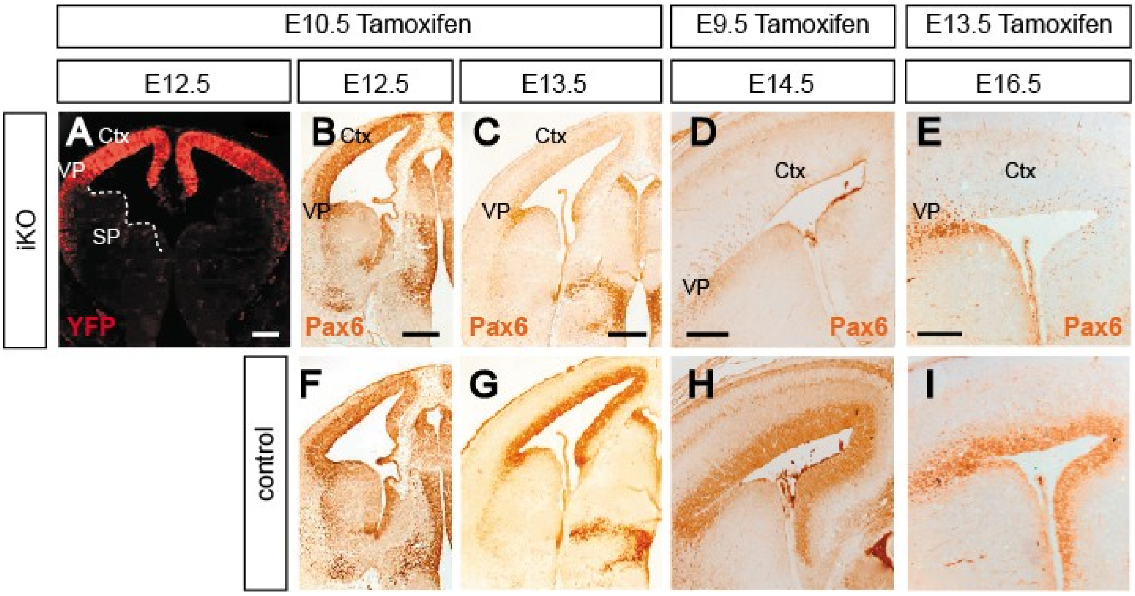

Figure S2. **Timing of Pax6 deletion from iKO embryos** (related to Fig. 2)**.** Expression of Pax6 protein in coronal sections from E12.5-E16.5 iKO and control embryos following tamoxifen administration on E10.5, E9.5 or E13.5; all embryos carried a YFP cre-reporter allele. (A) Expression of YFP specifically in the cortex (Ctx); SP = subpallium; VP = ventral pallium. (B,C,F,G) Specific loss of Pax6 protein from the cortex occurs 48-72h after tamoxifen administration; expression in the most ventral part of VP is spared since it is *Emx1*-negative. (D,H) Loss of Pax6 at E14.5 in iKO following tamoxifen at E9.5. (E,I) Loss of Pax6 at E16.5 in iKO following tamoxifen at E13.5. Scale bars = 100µm.

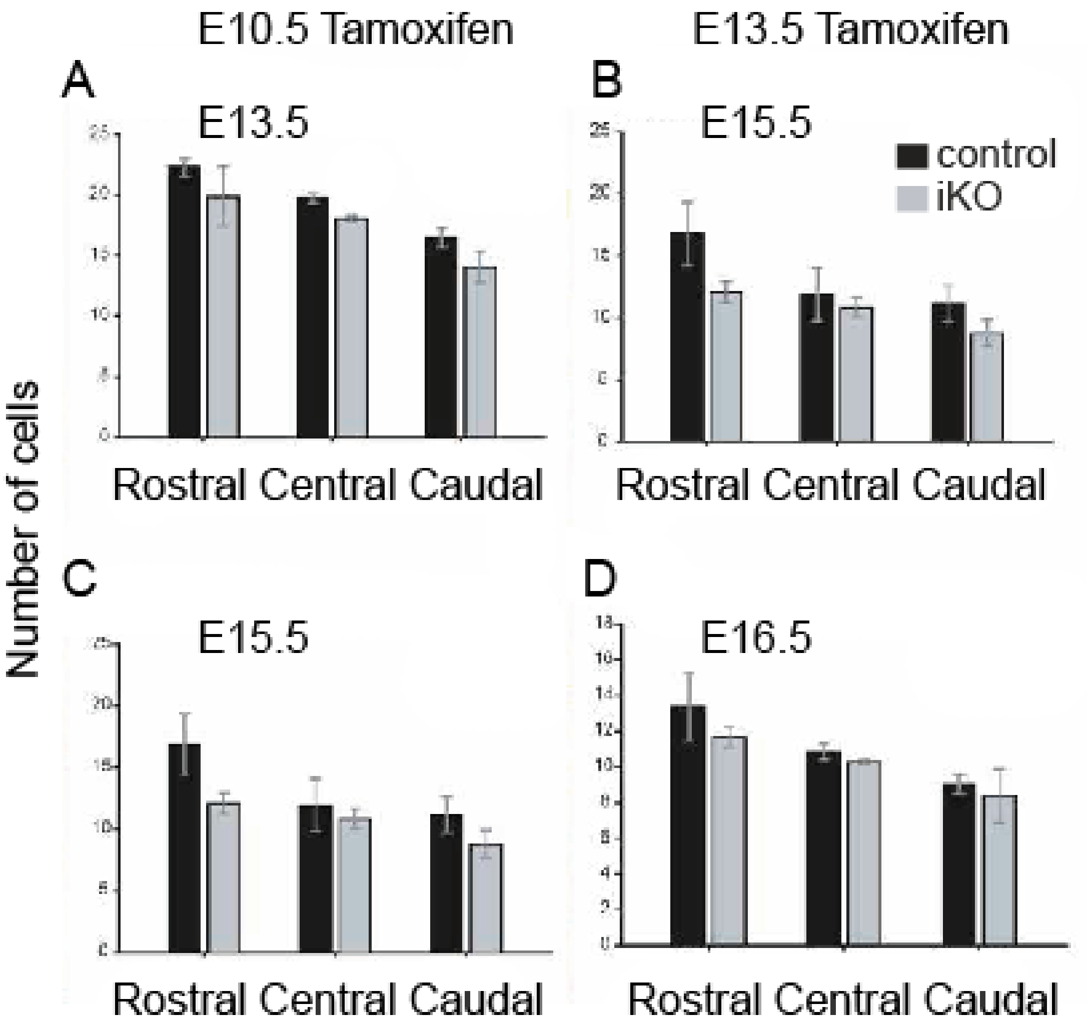

Figure S3. Counts of apical mitoses in conditional mutants (related to Fig. 2)**.** (A-D) Counts of PH3+ calls along the ventricular surface (apical; sampling boxes shown in Fig. 2) in E13.5, E15.5 and E16.5 embryos after tamoxifen at E10.5 or E13.5. Differences were not statistically significant. Error bars indicate s.e.m.

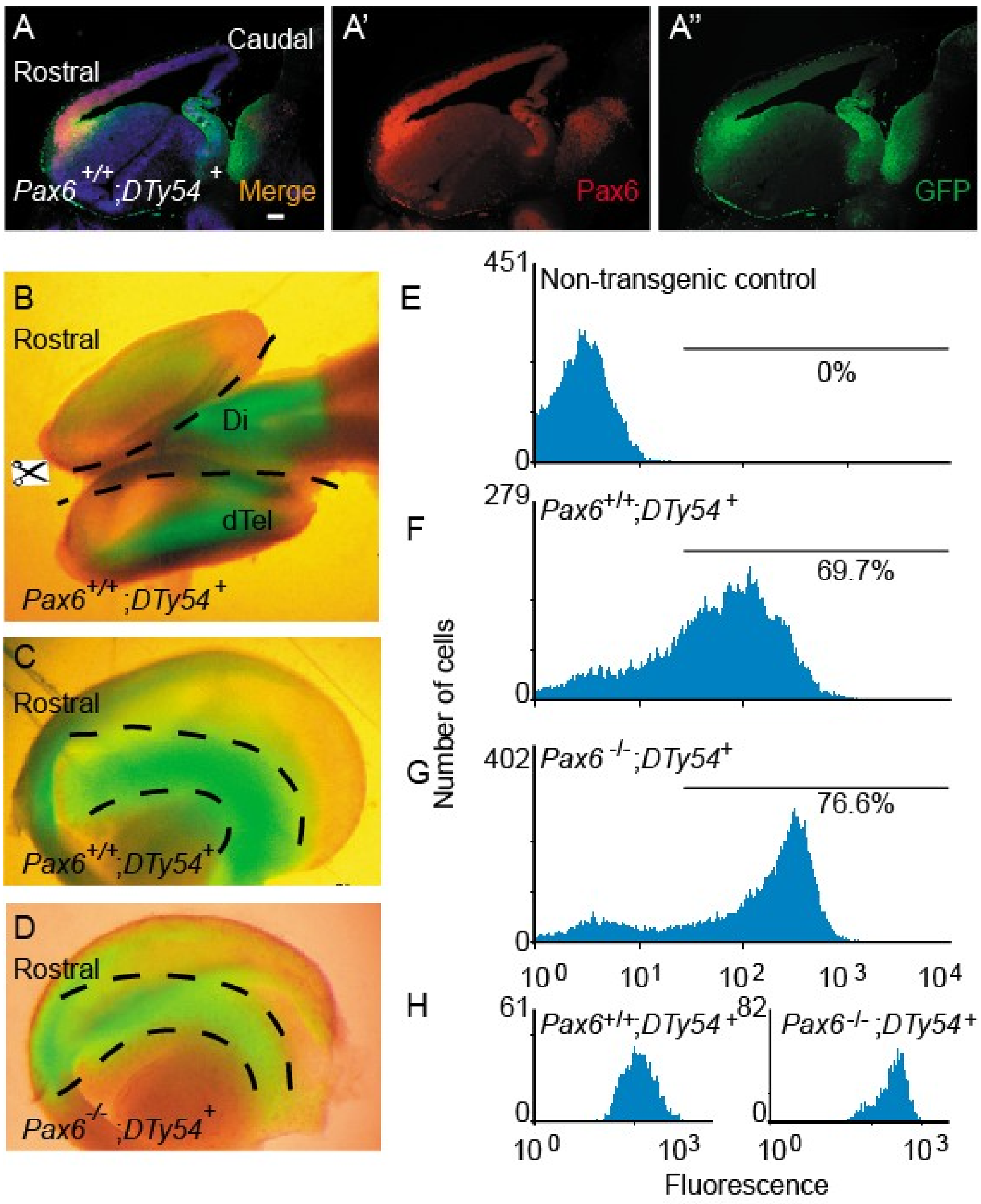

Figure S4. Separation of *Pax6*-expressing cells from *Pax6*$^{+/+}$ and *Pax6*$^{-/-}$ cortex using FACS (related to Fig. 3)**.** (A-A'') Parasagittal section of E12.5 *DTy54*$^{+}$ forebrain shows the close correspondence between levels of GFP and Pax6. Scale bar = 100µm. (B) A dorsal view of an E12.5 *DTy54*$^{+}$ brain showing GFP expression in the dorsal telencephalon (dTel) and diencephalon (Di); the telencephalic vesicles were removed as shown by broken lines. (C,D) *DTy54*$^{+}$ brains either wild-type or mutant for *Pax6* showing strongest GFP expression

laterally and rostrally (to the right); regions between the broken lines were dissociated for FACS. (E) Cells from non-transgenic controls were used to establish a gate for FACS of GFP-expressing cells from *DTy54*$^{+}$ brains. (F,G) Around 70-75% of cells from *DTy54*$^{+}$ wild-type or *Pax6*-mutant brains had fluorescence levels within the gate. (H) The histograms show the purity of the GFP-expressing populations after FACS.

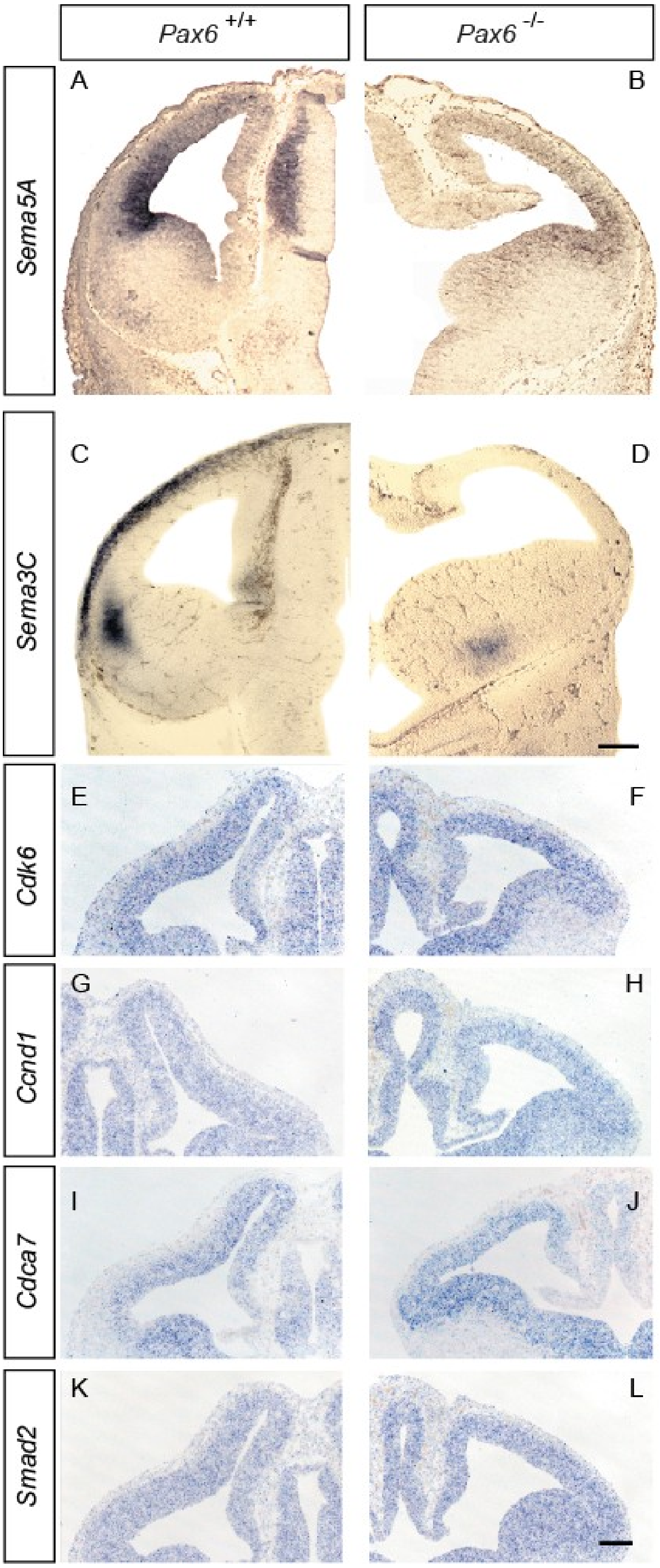

Figure S5. ***In situ*** **hybridisation experiments. (A-D) Quality-control of microarray results** (related to Fig. 3)**.** Previous studies of the expression of transcription factor genes in

Pax6$^{-/-}$ cortical progenitors showed up-regulation of *Gsh2*, *Dlx1*, *Lhx6* and *Ascl1* (*Mash1*) and down-regulation of *Ngn2*, *Neurod6* and *Tbr2* (Scardigli et al., 2003; Manuel and Price, 2005; Holm et al., 2007; Sansom et al., 2009; Georgala et al., 2011b). All of these were among the 20 most up-regulated and the 20 most down-regulated genes found in our analysis, supporting the validity of our approach. Panels A-D show *in situ* hybridizations testing expression of the signalling molecules *Sema5A* and *Sema3C* in E12.5 *Pax6*$^{-/-}$ cortex: since *Sema5A* is lost from the progenitor layer, this difference should have appeared in our microarray comparison, whereas *Sema3C* is lost from the superficial, postmitotic layer, so this difference should not have appeared. Indeed, in our microarray study *Sema5A* was significantly down-regulated (2.0-fold) whereas *Sema3C* was not. *Sema5A* and *Sema3C* probes were a kind gift from Andreas Püschel, Westfälische Wilhelms-Universität Münster, Germany. **(E-L) Expression of cell-cycle-related genes in E12.5 *Pax6*$^{+/+}$ and *Pax6*$^{-/-}$ cortex.** The *Ccnd1* probe was a kind gift from Peter Sicinski. (Whitehead Institute for Biomedical Research, Cambridge, Massachusetts, USA). Primers used to amplify the other probes were: *Cdk6* Forward: ACCGAGCCCCAGAAGT; *Cdk6* Reverse: AGCTCATGGGCATCCC; *Cdca7* Forward: TCTGAGAGCTCTGCAAACGA; *Cdca7* Reverse: AGCTGCAGTTGCAAATTCCT; *Smad2* Forward: AACCAGGTCTCTTGATGGCC; *Smad2* Reverse: GCCGTCTACAGTGAGCGAGG. All probes were digoxigenin-labelled. *In situ* hybridizations in A,B are on 10µm wax sections; those in C,D are on 100µm vibratome sections; those in E-L are on 10µm cryosections. Scale bars = 100µm.

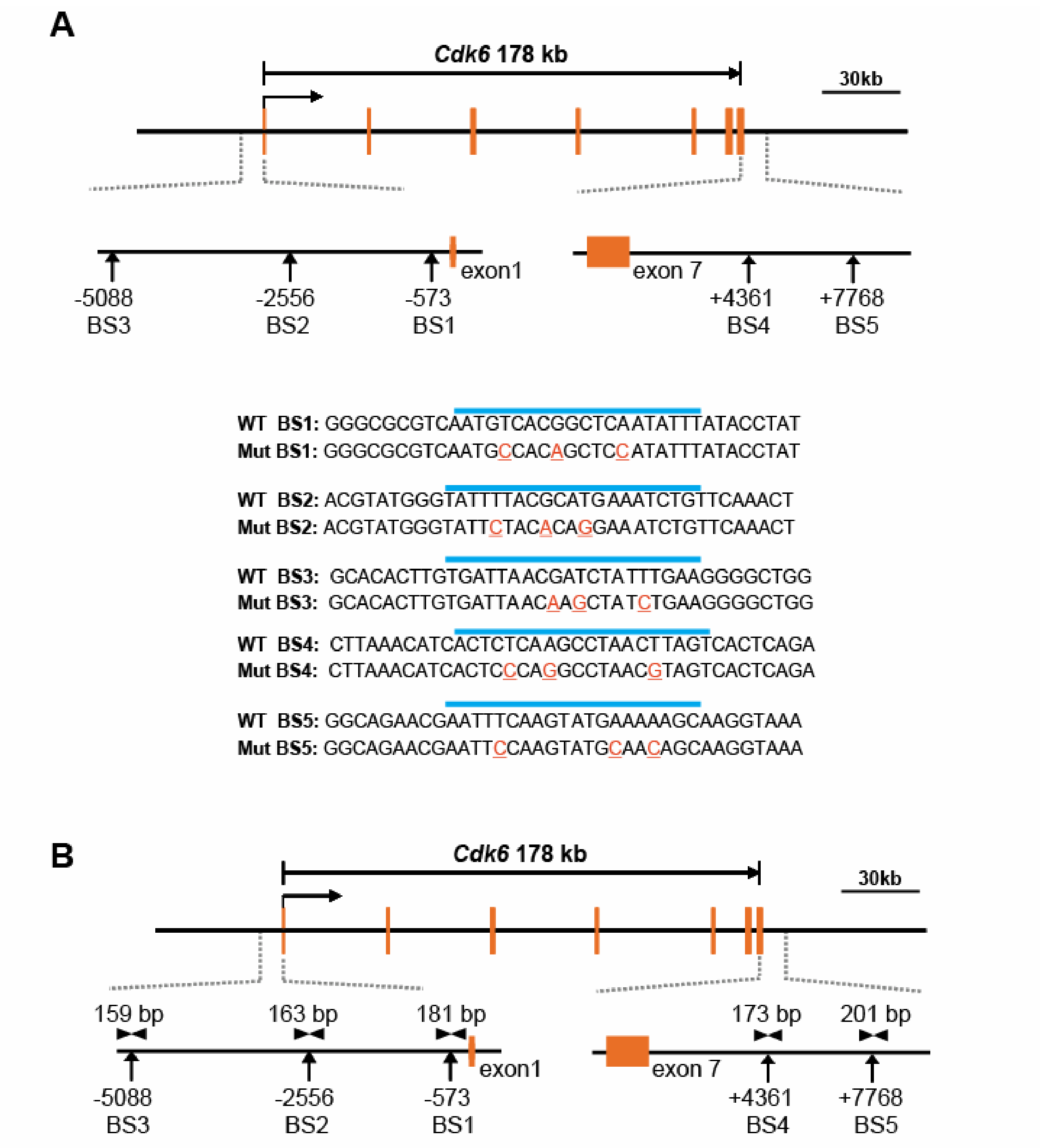

Locus Name	Primer Sequence (5'-3')	
	Forward	Reverse
BS1	CTGAAAGTAGAAAAGAAAGGAAGCAAT	ACCCGATGCTGCGAGTATG
BS2	GGGATTCCGTGCTGTAGAGTGT	GGAACTTGCACAGGGAAAATG
BS3	TCTGACTTTGGTTCAGTTTGTAAACA	AAAGCCAAAGAAGAGTTACAATGGA
BS4	GGACATACAACTAAAAACTTCCCTGAA	GCAAGGAGTTTTGCCATTTCTAA
BS5	TCTGGGTGCATAATTGCTCTCA	GAACCCAAAGCACCAAAACAG
Gab1	CGGTGACCTGACCTGTACTTACA	GCGGCGAAGTGGTTTGC
Syt8	GGGCCATGCCAACTTCAG	TCCTGCACACATGGAACCAT

Figure S6. (A) Details of five predicted Pax6 binding sites (BS1-5) around the *Cdk6* coding sequence (related to Figs. 4 and 5)**.** The positions of the wild-type sites are shown at the top. Numbers give the distance upstream or downstream from the nearest coding sequence to the first 5' nucleotide of each binding site. The sequence of oligonucleotides

used for EMSA analysis of each candidate binding site are given, together with mutant versions in which the predicted Pax6 binding site has been abolished (mutated based are in red and underlined). Blue lines mark the sequences identified using the position weight matrix (PWM) for the Pax6 consensus binding motif (P6CON; Epstein *et al.*, 1994a,b). **(B) Potential binding sites tested and primers used in ChIP experiments.** The diagram of the murine *Cdk6* locus shows the relative positions of predicted Pax6 binding sites (BS1-5; numbers give the distance upstream or downstream from the nearest coding sequence to the first 5' nucleotide of each binding site) and the sizes of the fragments (159-201 bp) spanning each site that were amplified by qPCR following ChIP.

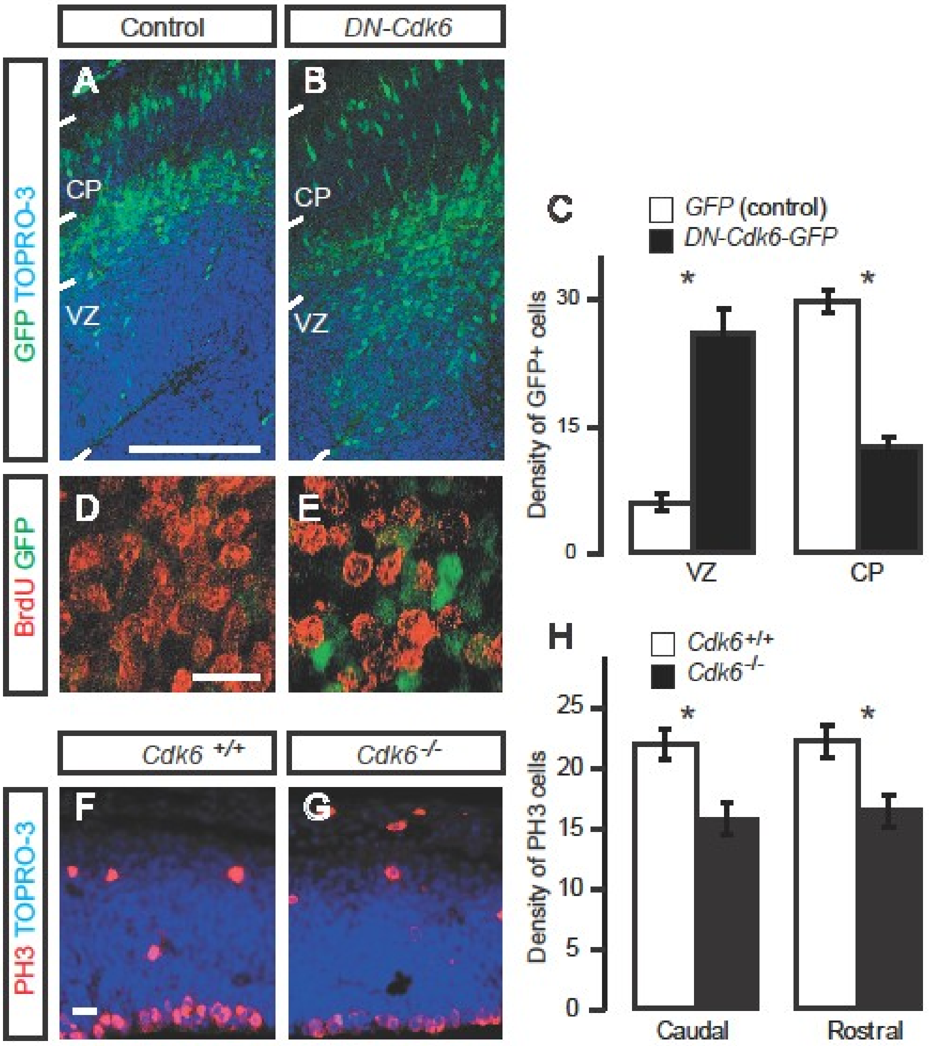

Figure S7. Inhibition or loss of Cdk6 slows cortical progenitor proliferation *in vivo* (related to Fig. 6)**.** (A-E) Analysis of the embryonic cortex 48h after electroporation of either control GFP-expressing or dominant-negative *Cdk6* (*DN-Cdk6*) and GFP-expressing constructs (used previously by Ferguson et al., 2000) into E12.5 wild-type embryos. (A,B) Many more *DN-Cdk6* GFP+ cells than control GFP+ cells remained in the ventricular zone (VZ). There were correspondingly lower densities of *DN-Cdk6* GFP+ cells in the cortical plate (CP). Scale bar,100µm. (C) Quantification of data from experiments illustrated in A and B. Values are means ± sems (n = 3 animals in each group) of GFP+ cell densities in 50µm (deep) × 200µm (wide) sampling areas in the cortical plate and 100µm × 100µm sampling areas in the ventricular zone; * $p < 0.00005$ (Student's t-test). (D, E) Images of the ventricular

zone: BrdU was given 1h before the embryos were sacrificed. In controls, very little GFP staining was detectable. In experimental animals, most *DN-Cdk6* GFP+ cells were negative for BrdU indicating that they were not undergoing cell division. Scale bar, 25µm. (F-H) Comparison of PH3 staining in *Cdk6*$^{+/+}$ and *Cdk6*$^{-/-}$ E12.5 embryos (Malumbres et al., 2004) showed significantly reduced densities (in 200µm wide sampling boxes) of apical PH3+ cells in *Cdk6*$^{-/-}$ mutants, in both rostral and caudal cortex. Scale bar, 10µm. (H) Graph shows means ± sems (n = 3 animals in each group). * $p < 0.05$ (Student's t-test).

Table S1. Primers for qRT-PCR (related to Fig. 3)

**Gene**	**Primers**
Cdk6	5'- TAGTTTTCAGATGGCCCTTA -3'<br>5'- GTTGATCCACGTCTGAACTT -3'
Ccnd1	5'- AGGAGCAGAAGTGCGAAGAG- 3'<br>5'- CACAACTTCTCGGCAGTCAA -3'
Cdca7	5'- GGAACGTCCATGCTTACTTG – 3'<br>5'- CACAACGTCGAGAACAAGAG- 3'
Smad2	5'- GCAGGAATTGAGCCACAGAG - 3'<br>5'- CGGAGAGCCTGTGTCCATAC – 3'
Mcm6	5'- GATTTCACAGGGGCACTGAT -3'<br>5'- AATGCTCTCAGCGGTCTGTT- 3'
Cdc6	5'- ACATCCTCGCTGCTGGTCTG – 3'<br>5'- GCGCTAACACTCATGAATCC- 3'
Lhx9	5'- GGACCTCAAACAGCTTGCTC - 3'<br>5'- CAGTGGGATTGGTCAGGTCT – 3'
Lhx6	5'- GCTAGCATTAGCGACCCTTG - 3'<br>5'- TGAAGCCATGGGTACCTCTC - 3'
Pax6/<br>*PAX6*	5'-TAGCGAAAAGCAACAGATG-3'<br>5'-TCTATTTCTTTGCAGCTTCC-3'

Table S2. PCR primers for cloning (related to Fig. 5)

Plasmid Name	Primer Sequence (5’-3’)
pBS1-luc	5’- AAAGAGCTCGCGTGCACTCTTGCTTCA – 3’<br>5’- AAACTCGAGTGCTGGCTTCAGGCTGCG – 3’
pBS2-luc	5’- GGTACCCCCTGGACCGCAGGTAAG – 3’<br>5’- GGTACCGGGCGAACGTTTTACTCCTT – 3’
pBS3-luc	5’- GGTACCGGCATAAAATTCCTCCCACA - 3’<br>5’- GGTACCACCAACCCAATGAAGCTGAC - 3’
pBS4-luc	5’- GGATCCGGTGGGGATGGAAGGTAGTT – 3’<br>5’- GGATCCTCATGGATAGTGAGGGGTGA – 3’
pBS5-luc	5’- GGATCCGGCATCAAGCATGTGATGAG – 3’<br>5’- GGATCCCTGGAGGGCACAGAGAAGAG – 3’

Table S3. PCR primers for mutagenesis (related to Fig. 5)

Plasmid Name	Primer Sequence (5’-3’)
pBS1mut-luc FWD	GAGAACAATAATATAGGTATAAATATGGAGCTGTGGCATT GACGCGCCCGG
pBS1mut-luc REV	CCGGGCGCGTCAATGCCACAGCTCCATATTTATACCTATA TTATTGTTCTC
pBS2mut-luc FWD	TCCTATGCCAGTTTGAACAGATTTCCTGTGTAGAATACCC ATACGTTCACTTTTCATC
pBS2mut-luc REV	GATGAAAAGTGAACGTATGGGTATTCTACACAGGAAATCT GTTCAAACTGGCATAGGA
pBS4mut-luc FWD	TGCTTTTTAGCTAGGTTCTGAGTGACTACGTTAGGCCTGGG AGTGATGTTTAAGC
pBS4mut-luc REV	GCTTAAACATCACTCCCAGGCCTAACGTAGTCACTCAGAA CCTAGCTAAAAGCA
